# Development and Characterization of Novel FAP-Targeted Theranostic Pairs: A Bench-to-Bedside Study

**DOI:** 10.34133/research.0282

**Published:** 2023-11-28

**Authors:** Wei Huang, Yizhen Pang, Qiufang Liu, Chenyi Liang, Shuxian An, Qianyun Wu, You Zhang, Gang Huang, Haojun Chen, Jianjun Liu, Weijun Wei

**Affiliations:** ^1^ Department of Nuclear Medicine, Institute of Clinical Nuclear Medicine, Renji Hospital, School of Medicine, Shanghai Jiao Tong University, Shanghai 200127, China.; ^2^ Department of Nuclear Medicine and Minnan PET Center, Xiamen Cancer Center, Xiamen Key Laboratory of Radiation Oncology, The First Affiliated Hospital of Xiamen University, School of Medicine, Xiamen University, Xiamen 361003, China.; ^3^ Department of Nuclear Medicine, Fudan University Shanghai Cancer Center, Fudan University, Shanghai 200032, China.

## Abstract

Fibroblast activation protein (FAP) is among the most popular targets in nuclear medicine imaging and cancer theranostics. Several small-molecule moieties (FAPI-04, FAPI-46, etc.) are used for developing FAP-targeted theranostic agents. Nonetheless, the circulation time of FAP inhibitors is relatively short, resulting in rapid clearance via kidneys, low tumor uptake, and associated unsatisfactory treatment efficacy. To address the existing drawbacks, we engineered 3 peptides named FD1, FD2, and FD3 with different circulation times through solid-phase peptide synthesis. All the 3 reported peptides bind to human and murine FAP with single-digit nanomolar affinity measured by surface plasmon resonance. The diagnostic and therapeutic potential of the agents labeled with ^68^Ga and ^177^Lu was assessed in several tumor models exhibiting different levels of FAP expression. While radiolabeled FD1 was rapidly excreted from kidneys, radiolabeled FD2/FD3 have significantly prolonged circulation, increased tumor uptake, and decreased kidney accumulation. Our findings indicated that [^68^Ga]Ga-DOTA-FD1 positron emission tomography (PET) effectively detected FAP dynamics, whereas [^177^Lu]Lu-DOTA-FD2 and [^177^Lu]Lu-DOTA-FD3 exhibited remarkable therapeutic efficacy in FAP-overexpressing tumor models, including pancreatic cancer cell models characterized by abundant stroma. Moreover, a pilot translational investigation demonstrated that [^68^Ga]Ga-DOTA-FD1 had the capability to identify both primary and metastatic tumors with precision and distinction. In summary, we developed [^68^Ga]Ga-DOTA-FD1 for same-day PET imaging of FAP dynamics and [^177^Lu]Lu-DOTA-FD2 and [^177^Lu]Lu-DOTA-FD3 for effective radioligand therapy of FAP-overexpressing tumors.

## Introduction

Tumor imaging and therapy have gained significant interest in recent years due to the focus on biomarkers within the tumor microenvironment [1,2]. Tumor development and progression are regulated by the symbiotic interaction between tumor cells and the surrounding stroma in solid tumors [3]. The tumor microenvironment accounts for a substantial portion of the tumor [4]. According to reports, the tumor microenvironment can comprise up to 80% of the tumor mass in cases of pancreatic ductal carcinoma [5]. Abundant components of the tumor microenvironment are cancer-associated fibroblasts (CAFs), which are extensively present in the stroma of various tumors including pancreatic, breast, and colon cancers [6,7]. The role of CAFs in promoting tumor growth, invasion, angiogenesis, and modification of the extracellular matrix has been extensively documented [8–10].

Fibroblast activation protein (FAP), a glycoprotein of the dipeptidyl peptidase family [11,12], is abundantly expressed in CAFs of numerous epithelial tumors, while its presence in normal tissues is limited. This characteristic makes it a promising universal target for the diagnosis and treatment of different types of cancer [13,14]. In the tumor microenvironment of most epithelial malignancies and several mesenchymal origin malignancies, particularly sarcoma and mesothelioma, CAFs exhibit an uncertain presence of FAP on their cell surface [13]. Following initial trials with a FAP-specific antibody probe (^131^I-sibrotuzumab) for single photon emission computed tomography (SPECT) imaging [15], a team of researchers at the University of Antwerp successfully created a range of FAP inhibitors (FAPIs) using quinoline-based small molecules [16]. The pharmacokinetics of these FAPIs were additionally assessed in preclinical models to develop positron emission tomography/computed tomography (PET/CT) radiotracers that can be used in practice [4,17,18]. Among them, FAPI-04/46 showed encouraging diagnostic efficacies in a broad spectrum of preclinical tumor models and human subjects, but their therapeutic efficacy was limited by the relatively short tumor retention time [19–21]. Another novel FAPI derivative, FAP-2286, exploits a cyclic peptide as the binding unit, possesses a longer tumor retention time than traditional FAPIs (FAP-04/46), and has achieved promising diagnostic and therapeutic results in both preclinical models and clinical studies [22–24]. Recent years have witnessed a growth in research on FAP-targeted molecular agents for tumor diagnosis and therapy. In addition, several chemical optimization strategies, such as albumin binding and polymerization, have been reported to prolong the retention and increase tumor uptake of these radioligands [25–28].

Increased renal radioactivity of radiolabeled small-molecule or peptide substances eliminated through the kidneys could be linked to endocytosis and transcellular transportation facilitated by macrophage proteins, or hydrolysis of lysosomal proteins following glomerular filtration and renal reabsorption [29–31]. Arano and colleagues presented a successful approach for decreasing renal absorption of radiopharmaceuticals by including particular sequences such as Met-Val-Lys (MVK) that can be identified and cleaved between Met-Val residues through a metalloendopeptidase enzyme, which is highly abundant in the lining of the renal brush border membrane, especially in the proximal tubule [32–34].

During this research, we successfully created 3 variations (FD1, FD2, and FD3) of FAP-2286 that possess distinct characteristics. FD1 was linked to the MVK sequence to optimize pharmacokinetic properties expecting lower radioactivity in the kidneys. Both FD2 and FD3 contained albumin-binding domains to improve tumor retention and therapeutic efficacies, with the latter bearing additional MVK sequence to accelerate clearance of the radioligand when circulated to the kidneys. To evaluate the effectiveness of diagnosis and antitumor treatment in various preclinical models, the 3 derivatives were labeled using ^68^Ga and ^177^Lu. Additionally, a pilot clinical study investigated the diagnostic effectiveness of [^68^Ga]Ga-DOTA-FD1 in individuals with different types of tumors.

## Results

### Characterization of FD1, FD2, and FD3

**Fig. 1.**
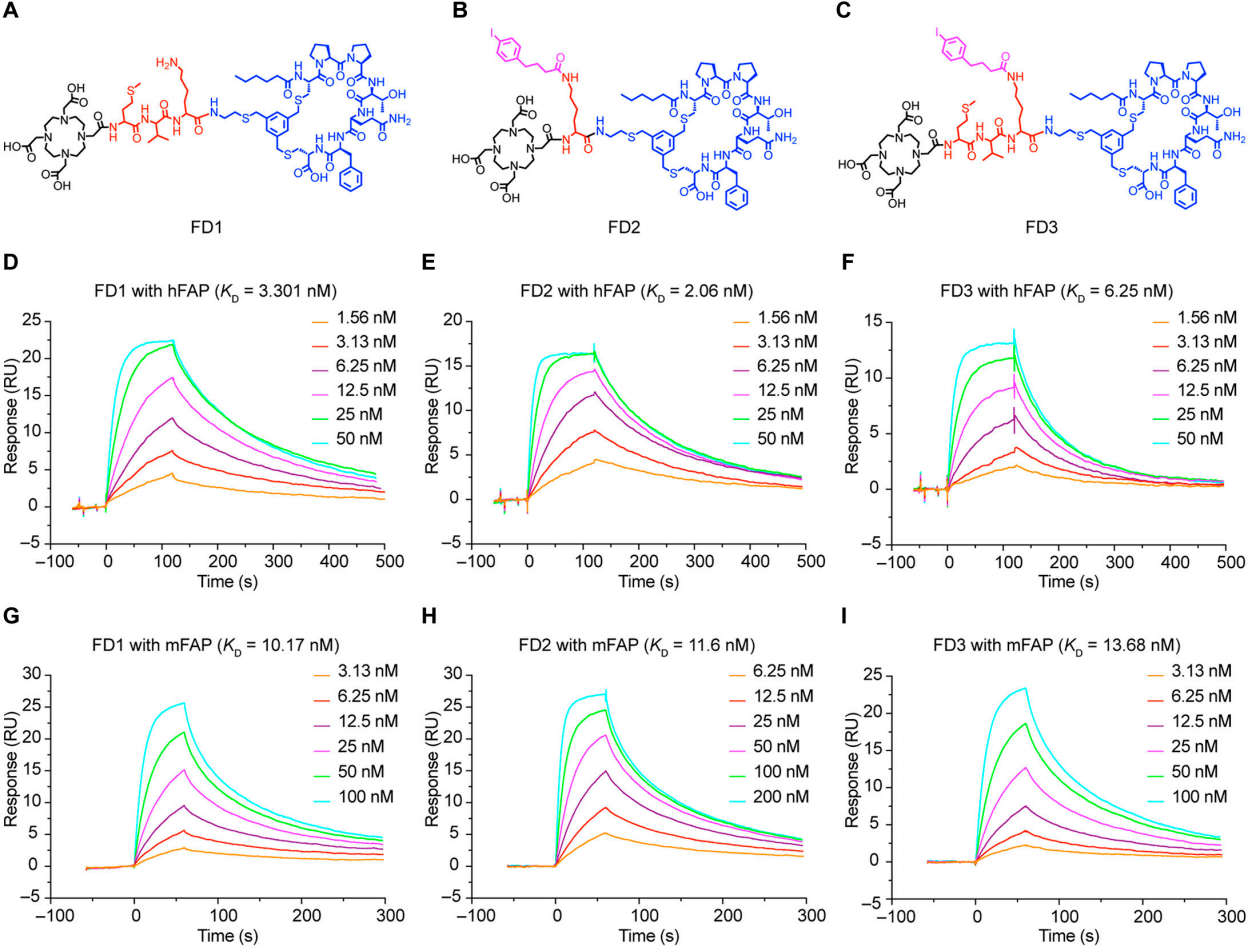
The structures of FD1/FD2/FD3 and the binding affinity of the precursors interacting with immobilized recombinant human and murine FAP protein were tested by SPR. (A to C) The structures of FD1, FD2, and FD3. (D to F) FD1, FD2, and FD3 reactive curves with recombinant human FAP protein. (G to I) FD1, FD2, and FD3 reactive curves with recombinant murine FAP protein.

FD1, FD2, and FD3 are 3 FAP-binding cyclic peptides derived from the parental peptide FAP-2286. The chemical structures of FD1/FD2/FD3 are shown in Fig. 1A to C. An MVK sequence was inserted into FAP-2286 to produce FD1 aiming to potentially reduce kidney accumulation. FD2 was created by incorporating a 4-(p-iodophenyl) butyric acid moiety into FAP-2286, with the objective of extending in vivo circulation and enhancing tumor uptake. The MVK sequence and 4-(p-iodophenyl) butyric acid moiety were introduced into FAP-2286 in tandem to produce FD3. All 3 FAP-2286 derivatives have DOTA chelators for different radionuclides labeling. After constructing the 3 peptides, the purity of the peptides determined by high-performance liquid chromatography (HPLC) was >95% (Fig. S1). Surface plasmon resonance (SPR) was utilized to evaluate the interaction between FD1, FD2, and FD3 with recombinant human and murine FAP protein (Fig. 1D to I). The equilibrium dissociation constant (*K*_D_) value of the 3 peptides binding to the 2 recombinant proteins exhibited a robust affinity for FAP, being in the nanomolar (nM) range. The *K*_D_ value for FD1, FD2, and FD3 when interacting with human FAP protein was 3.301 nM, 2.06 nM, and 6.25 nM (Fig. 1D to F), respectively, whereas the *K*_D_ value with recombinant murine FAP protein was 10.17 nM, 11.6 nM, and 13.68 nM (Fig. 1G to I). The binding of the peptides to murine FAP guarantees thorough evaluation of the diagnostic and therapeutic efficacies in non-humanized tumor-bearing mice models.

### [^68^Ga]Ga-DOTA-FD1 PET imaging in various tumor models

**Fig. 2.**
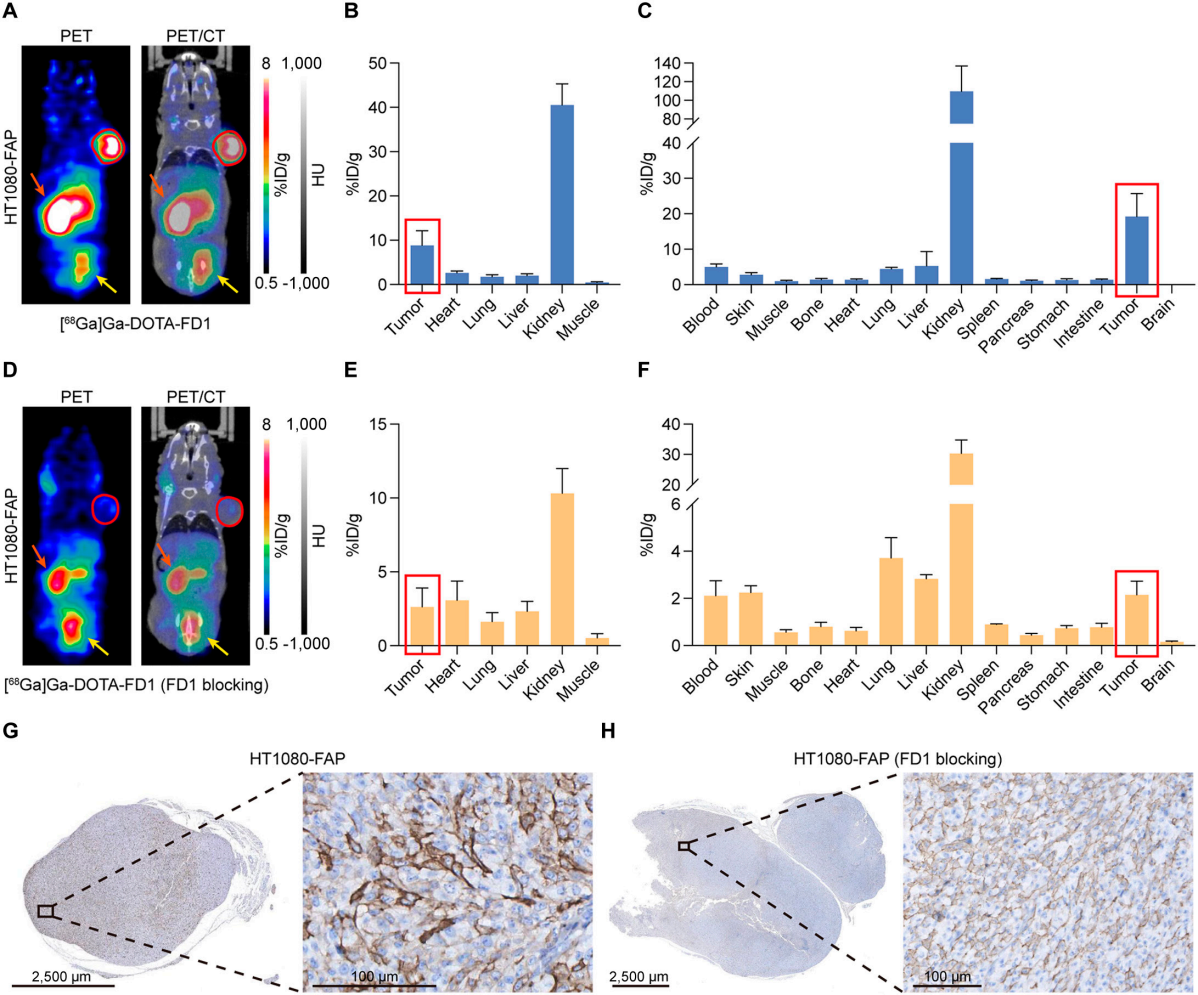
[^68^Ga]Ga-DOTA-FD1 PET imaging in HT1080-FAP tumor models. (A, D) Coronal images of [^68^Ga]Ga-DOTA-FD1 PET/CT in the subcutaneous HT1080-FAP models without (A, *n* = 4) or with (D, *n* = 4) excess FD1 blocking. Tumors were delineated by red circles. The kidney and bladder are shown by orange and yellow arrows, respectively. (B, E) Quantitative analysis of region of interest (ROI) data showing the uptake and distribution of the tracer in terms of %ID/g. (C, F) The ex vivo biodistribution data showing the detailed distribution patterns of [^68^Ga]Ga-DOTA-FD1 in the tumors and major tissues/organs of the HT1080-FAP group (C, *n* = 3) and FD1 blocking HT1080-FAP group (F, *n* = 3). (G, H) IHC staining results of the fixed tumor tissues from the 2 groups.

We stained several fixed tumor samples from our laboratory using an anti-FAP antibody (Fig. S2). The tumor tissues comprised BxPC-3 (pancreatic adenocarcinoma), Huh-7 (hepatocellular carcinoma), MCF-7 (breast cancer), SKOV-3 (ovarian cancer), No. 490 PDX (gastric cancer), and LS174T (colorectal adenocarcinoma). For molecular imaging, tumor models with different levels of FAP expression were developed using the following cell lines: BxPC-3 (human pancreatic cancer), Panc-02 (murine pancreatic cancer), HT-1080 (human fibrosarcoma), and HT1080-FAP (HT1080 with stable overexpression of human FAP). The labeling of FD1 with ^68^Ga ([^68^Ga]Ga-DOTA-FD1) was efficient and rapid with radiochemical purity (RCP) exceeding 99% upon completion of the reaction, thereby obviating the necessity for additional purification (Fig. S3A). Static microPET/CT imaging of BxPC-3- and Panc-02-bearing mice was performed 1 h after tracer injection. Representative images showed slight uptake at the tumor site (Fig. S4A and D). The remaining unbound probe was quickly eliminated via the urinary system. The analysis of the region of interest (ROI) revealed low uptake in BxPC-3 (0.96 ± 0.07%ID/g, *n* = 3) and Panc-02 (0.92 ± 0.22%ID/g, *n* = 4, Fig. S4B and E) tumors. The findings from ex vivo biodistribution investigations following imaging were in line with the PET-semiquantitative outcomes (Fig. S4C and F). We additionally interrogated the diagnostic efficacy of [^68^Ga]Ga-DOTA-FD1 in HT-1080 tumor models 30 min post-injection (p.i.) of the tracer (Fig. S4G). The HT-1080 tumor consistently exhibited low uptake, specifically 1.97 ± 0.45%ID/g (*n* = 3, Fig. S4H) in ROI analysis and 1.02 ± 0.41%ID/g (*n* = 3, Fig. S4I) in the biodistribution study. Contrastingly, sharp and rapid uptake of [^68^Ga]Ga-DOTA-FD1 was observed at the tumor site of HT1080-FAP models, as depicted in Fig. 2A. The quantitative tumor uptake revealed by ROI analysis (8.85 ± 3.30%ID/g, *n* = 4; Fig. 2B) and biodistribution study (19.20 ± 6.51%ID/g, *n* = 3; Fig. 2C) was significantly greater than that in other tumor types (ROI: *P* < 0.01 in BxPC-3 and Panc-2 models, *P* < 0.05 in HT-1080 models; biodistribution: *P* < 0.01 in all the 3 models). In order to confirm the binding specificity to FAP, [^68^Ga]Ga-DOTA-FD1 and excess unlabeled FD1 (750 μg) were co-injected into HT1080-FAP tumor models, in which the cold FD1 competitively bound to and saturated the target. Upon excessive FD1 blocking, a significant drop in tumor uptake and kidney accumulation was observed (Fig. 2D). Head-to-head comparison of the ROI data showed significant decrease in tumor uptake upon FD1 blocking (8.85 ± 3.30%ID/g vs. 2.63 ± 1.28%ID/g, *n* = 4 per group, *P* < 0.05; Fig. 2B and E), which was further verified by comparing the biodistribution results (19.20 ± 6.51%ID/g vs. 2.15 ± 0.58%ID/g, *n* = 3 per group, *P* < 0.01; Fig. 2C and F). Interestingly, FD1 blocking also significantly decreased kidney accumulation of [^68^Ga]Ga-DOTA-FD1 (ROI analysis: 40.58 ± 4.76%ID/g vs. 10.33 ± 1.67%ID/g, *n* = 4 per group, *P* < 0.01; biodistribution analysis: 109.81 ± 27.14%ID/g vs. 30.34 ± 4.45%ID/g, *n* = 3 per group, *P* < 0.01). This reduction was partially attributed to the blockade of [^68^Ga]Ga-DOTA-FD1 accumulation and reabsorption in the kidneys. Subsequent immunohistochemical (IHC) staining of the resected tumors confirmed high FAP expression in HT1080-FAP tumors (Fig. 2G and H) and low FAP expression in BxPC-3, Panc-02, and HT-1080 tumors (Fig. S5A, D, and G).

### Multi-time PET imaging with [^68^Ga]Ga-DOTA-FD2 and [^68^Ga]Ga-DOTA-FD3

[^68^Ga]Ga-DOTA-FD2 and [^68^Ga]Ga-DOTA-FD3 were radiolabeled at an average specific activity of around 1.85 GBq/μmol, with the corresponding RCP of 98% and 94% without purification (Fig. S3B and C). Three xenograft tumor models, including BxPC-3, Panc-02, and HT-1080, were utilized to investigate the in vivo distribution pattern of [^68^Ga]Ga-DOTA-FD2 and [^68^Ga]Ga-DOTA-FD3 at multi-time points (30 min, 2 h, and 4 h). This was done considering the anticipated prolonged circulation duration of FD2 and FD3. The microPET/CT images showed a slight increase at the tumor site within 4 h (Figs. S6A to S8A and S6D to S8D). Similarly, we outlined ROIs and quantified the radioactivity absorbed in the tumor, heart (blood pool), lung, liver, kidney, and muscle. The average uptake of [^68^Ga]Ga-DOTA-FD2 and [^68^Ga]Ga-DOTA-FD3 in BxPC-3 tumor was 7.67 ± 0.38%ID/g (*n* = 3) and 7.07 ± 0.45%ID/g (*n* = 3), that in Panc-02 tumor was 6.90 ± 0.51%ID/g (*n* = 4) and 7.03 ± 1.00%ID/g (*n* = 4), and that in HT-1080 was 8.10 ± 0.52%ID/g (*n* = 3) and 9.97 ± 0.42%ID/g (*n* = 3) at 4 h p.i., respectively. Over the 4-h imaging period, the radioactivity uptake in the heart (blood pool), lung, liver, and kidney diminished. Notably, the accumulation of radioactivity in the blood pool remained significantly higher than that in the other tissues/organs. Muscle uptake remained stable at a low level at the 3 imaging time points (Figs. S6B to S8B and S6E to S8E). The HT1080-FAP model showed a comparable distribution pattern following the injection of [^68^Ga]Ga-DOTA-FD2. The tumor uptake of [^68^Ga]Ga-DOTA-FD2 gradually increased with the corresponding uptake value of 6.63 ± 0.64 at 30 min, 8.23 ± 2.61 at 2 h, and 9.37 ± 0.61%ID/g at 4 h, respectively. Tracer distribution pattern in other organs was consistent with changes in the above 3 tumor models with low FAP expression (Fig. S8G and H). The biodistribution data of the 2 agents in all tumor models were collected at 4.5 h p.i. (Figs. S6C to S8C and S6F to S8F; Fig. S8I). In general, the blood pool showed the highest radioactivity retention in all the 4 models, which is in line with the ROI quantitative analysis data. Meanwhile, tumor uptake of HT1080-FAP models was higher than the other 3 tumor models after analyzing the biodistribution data (HT1080-FAP vs. BxPC-3, *P* < 0.05; HT1080-FAP vs. Panc-02, *P* < 0.01; HT1080-FAP vs. HT-1080, 11.89 ± 1.83%ID/g vs. 10.38 ± 1.84%ID/g, *P* > 0.05). High and low expression of FAP was confirmed in representative fixed tumor tissues on IHC staining (Fig. S5B, C, E, F, and H to J). The data together demonstrated improved pharmacokinetics of FD2 and FD3, but therapeutic radionuclides of relatively long high-life are needed to match the circulation time and unleash the therapeutic potential.

### Development and characterization of ^177^Lu-labeled FD1/FD2/FD3

**Fig. 3.**
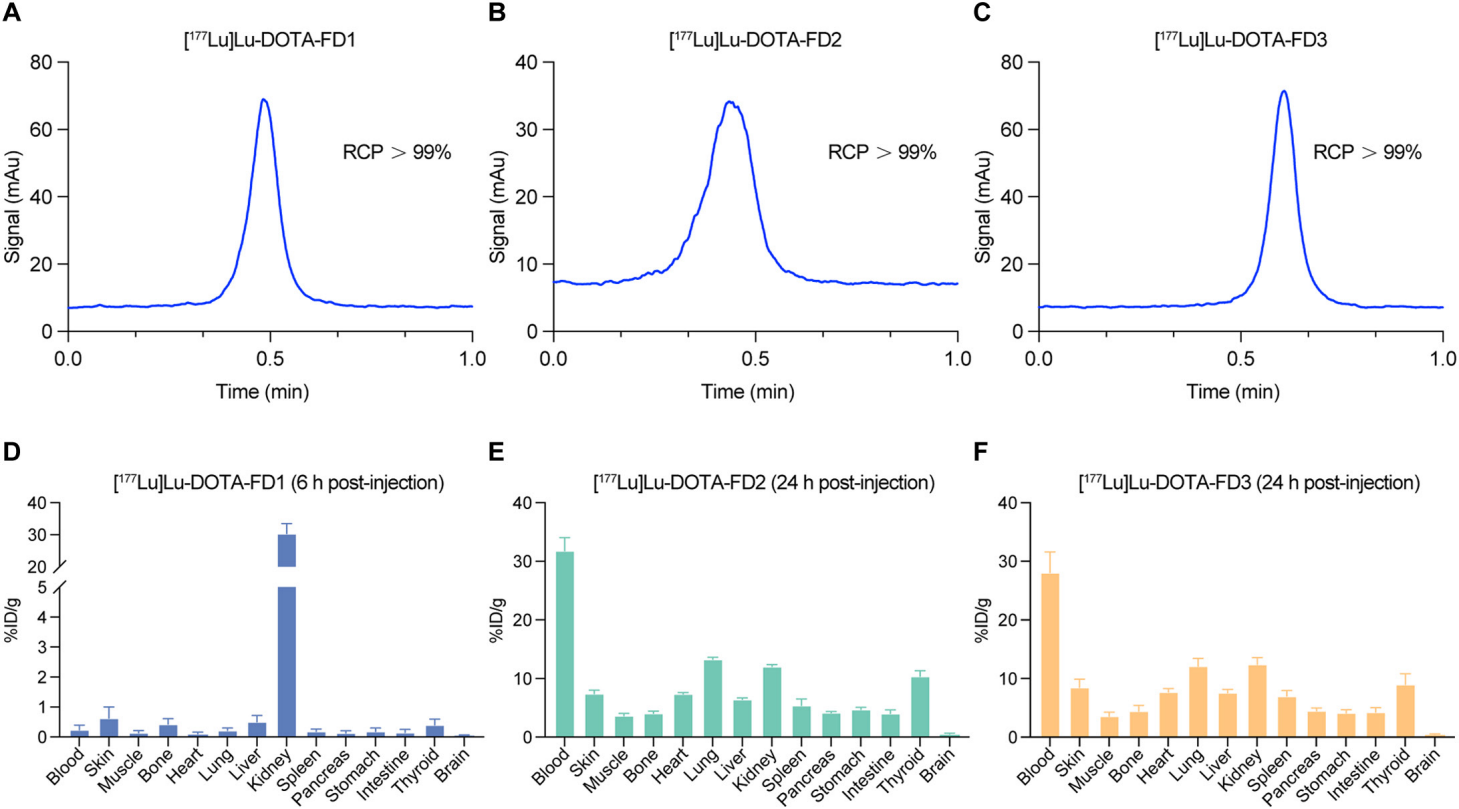
Characterization of [^177^Lu]Lu-DOTA-FD1, [^177^Lu]Lu-DOTA-FD2, and [^177^Lu]Lu-DOTA-FD3. The radiochemical purity (RCP) of [^177^Lu]Lu-DOTA-FD1 (A), [^177^Lu]Lu-DOTA-FD2 (B), and [^177^Lu]Lu-DOTA-FD3 (C). The radioactive biodistribution of [^177^Lu]Lu-DOTA-FD1 at 6 h post-injection (D, *n* = 4), [^177^Lu]Lu-DOTA-FD2 at 24 h post-injection (E, *n* = 3), and [^177^Lu]Lu-DOTA-FD3 at 24 h post-injection (F, *n* = 3) in tumor-free nude mice.

With the above data in hand, we proceeded to develop ^177^Lu-labeled peptides for FAP-targeted therapies. The RCP of all ^177^Lu-labeled peptides was >99% without further purification (Fig. 3A to C). [^177^Lu]Lu-DOTA-FD1, [^177^Lu]Lu-DOTA-FD2, and [^177^Lu]Lu-DOTA-FD3 were stable in fetal bovine serum (FBS) and phosphate-buffered saline (PBS) for at least 24 h, with no significant demetallation noticed (Fig. S9). After the successful development of ^177^Lu-labeled radioligands, the in vivo biodistribution of these 3 radioligands in tumor-free nude mice at a certain time point was determined ([^177^Lu]Lu-DOTA-FD1 group at 6 h p.i., and [^177^Lu]Lu-DOTA-FD2 and [^177^Lu]Lu-DOTA-FD3 groups at 24 h p.i.). The biodistribution data of [^177^Lu]Lu-DOTA-FD1 were acquired at 6 h p.i. and showed lower radioactivity uptake in all collected samples except the kidneys (30.40 ± 3.10%ID/g, *n* = 4, Fig. 3D), confirming the short in vivo circulation time and rapid urinary clearance of FD1 irrespective of labeling radiometals. At 24 h p.i., [^177^Lu]Lu-DOTA-FD2 and [^177^Lu]Lu-DOTA-FD3 exhibited continuous accumulation in the blood pool, reaching 31.82 ± 2.24%ID/g (*n* = 3, Fig. 3E) and 28.09 ± 3.52%ID/g (*n* = 3, Fig. 3F), respectively. The prolonged circulation of the 2 radioligands resulted in higher radioactivity accumulation in organs other than kidneys when compared to [^177^Lu]Lu-DOTA-FD1. We conducted further assessments on the distribution and accumulation of [^177^Lu]Lu-DOTA-FD3 in HT1080-FAP tumor models at 45 h and 90 h p.i. Similarly, [^177^Lu]Lu-DOTA-FD3 was mainly retained in the blood pool at 45 h p.i. (16.34 ± 2.50%ID/g, *n* = 4) and gradually decreased at 90 h p.i. (11.46 ± 1.54%ID/g, *n* = 3, Fig. S10). The tumor uptake of [^177^Lu]Lu-DOTA-FD3 gradually increased from 10.52 ± 1.02%ID/g at 45 h (*n* = 4) to 12.36 ± 3.12%ID/g at 90 h (*n* = 3). To conclude, both [^177^Lu]Lu-DOTA-FD2 and [^177^Lu]Lu-DOTA-FD3 have improved pharmacokinetics, making them appropriate for subsequent FAP-targeted therapies.

### FAP-targeted radioligand therapy in HT1080-FAP tumor models

**Fig. 4.**
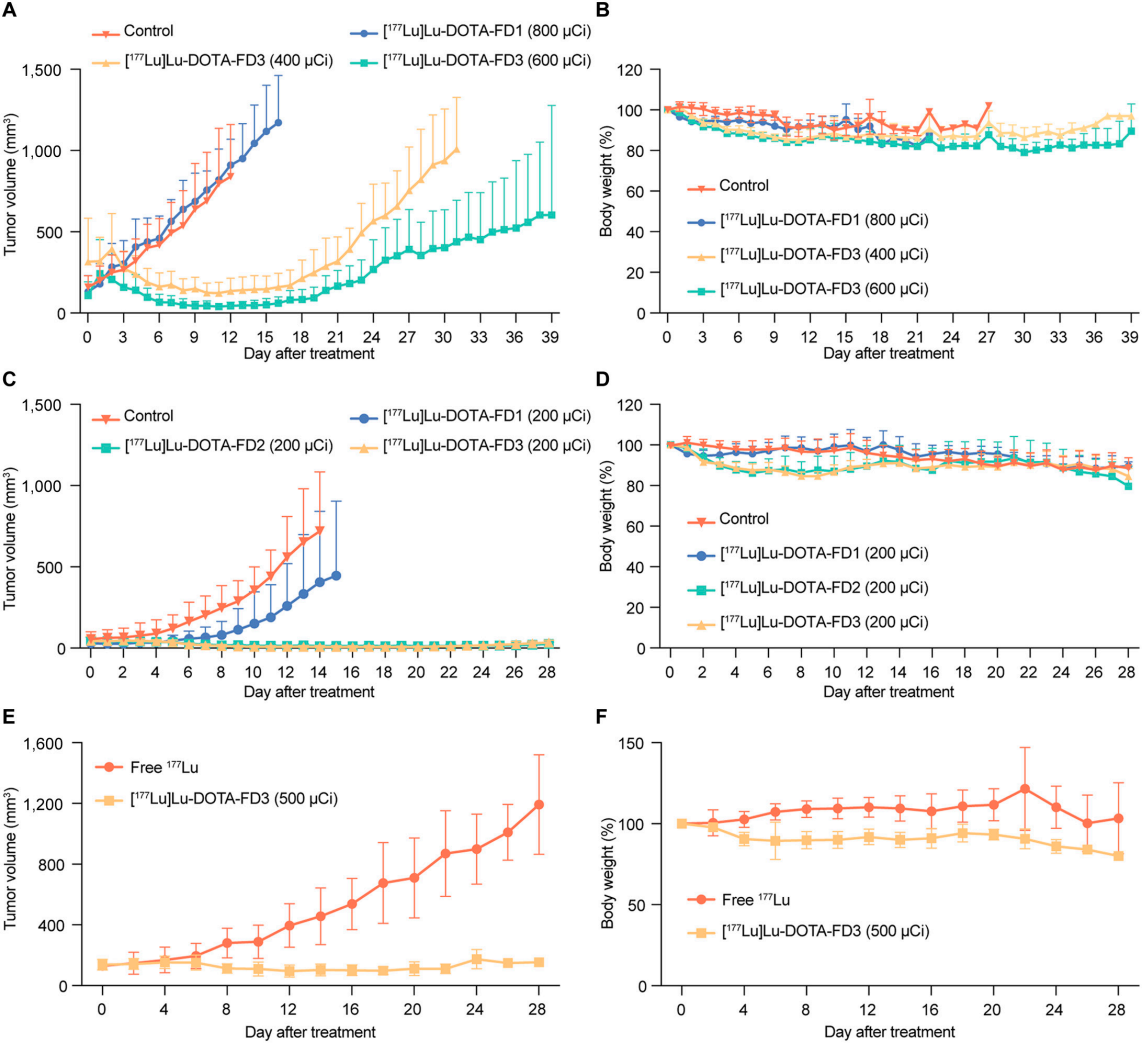
FAP-targeted radioligand therapy in HT1080-FAP and T3M-4 models. (A) Monitoring of the tumor volume in HT1080-FAP models with an average tumor volume of 180 mm^3^ at the onset of the treatments (*n* = 5 per group). (B) Changes in mice’s body weight are presented as the percentage of initial body weight (*n* = 5 per group). Changes in tumor volume (C) and body weight (D) in HT1080-FAP models with an average tumor volume of 50 mm^3^ at the onset of the treatments (*n* = 6 per group). Changes in tumor volume (E) and body weight (F) in the T3M-4 pancreatic cancer cell models with abundant stroma (*n* = 7 per group).

We first investigated the therapeutic efficacies [^177^Lu]Lu-DOTA-FD1 and [^177^Lu]Lu-DOTA-FD3 in the HT1080-FAP tumor models with an average tumor volume of 180 mm^3^. Tumor-bearing mice were divided into 4 groups: control group, 800 μCi [^177^Lu]Lu-DOTA-FD1 treatment group, 400 μCi [^177^Lu]Lu-DOTA-FD3 treatment group, and 600 μCi [^177^Lu]Lu-DOTA-FD3 treatment group. Tumor volume and body weight changes in the mice were monitored daily during the treatment. As illustrated in Fig. 4A, different antitumor efficacies of [^177^Lu]Lu-DOTA-FD1 and [^177^Lu]Lu-DOTA-FD3 were recorded. Mice were euthanized when the volume exceeded 1,500 mm^3^ or surface necrosis occurred. The tumor volume in the control group and [^177^Lu]Lu-DOTA-FD1 treatment group steadily increased. There was no difference in tumor volume between the [^177^Lu]Lu-DOTA-FD1 group and the control group. In comparison, the tumor volume of the [^177^Lu]Lu-DOTA-FD3 treatment group began to shrink from day 2 and the 600 μCi treatment group tumor showed a more thorough tumor suppressive effect than the 400 μCi treatment group. Significant differences in tumor volume were calculated in the 600 μCi/400 μCi [^177^Lu]Lu-DOTA-FD3 treatment groups compared with the [^177^Lu]Lu-DOTA-FD1 treatment group from day 6 and day 7, respectively (*P* = 0.030 and *P* = 0.017, respectively). However, the tumors in the [^177^Lu]Lu-DOTA-FD3 treatment groups relapsed from day 14 onward. More specifically, tumor volume in the 400 μCi [^177^Lu]Lu-DOTA-FD3 treatment group doubled at a rate close to that of the control and [^177^Lu]Lu-DOTA-FD1 treatment groups, whereas tumors in the 600 μCi [^177^Lu]Lu-DOTA-FD3 treatment group grew at a much slower pace. Mice in all the experimental groups had slight weight loss with the 600 μCi [^177^Lu]Lu-DOTA-FD3 treatment group losing more than the 400 μCi [^177^Lu]Lu-DOTA-FD3 treatment group (Fig. 4B).

Since single-dose administration of [^177^Lu]Lu-DOTA-FD1 and [^177^Lu]Lu-DOTA-FD3 failed to show sustained therapeutic efficacy for tumors of big size (180 mm^3^), we then initiated the treatment when the average tumor volume reached 50 mm^3^ with a lower given dose. Similarly, mice were randomly divided into 4 groups: control group, 200 μCi [^177^Lu]Lu-DOTA-FD1 treatment group, 200 μCi [^177^Lu]Lu-DOTA-FD2 treatment group, and 200 μCi [^177^Lu]Lu-DOTA-FD3 treatment group. In contrast to the control group, treatment with [^177^Lu]Lu-DOTA-FD2 and [^177^Lu]Lu-DOTA-FD3 led to significant differences in tumor volume from day 9 onward (*P* = 0.048 and *P* = 0.042, respectively). Both [^177^Lu]Lu-DOTA-FD2 and [^177^Lu]Lu-DOTA-FD3 treatment substantially inhibited the tumor growth of the tumors at the end of the study (Fig. 4C). More specifically, 1 of the 6 mice in the 200 μCi [^177^Lu]Lu-DOTA-FD2 treatment group and 3 out of the 6 mice in the 200 μCi [^177^Lu]Lu-DOTA-FD3 treatment acquired complete response (CR). Interestingly, all 4 CR tumors mentioned above suffered from recurrence despite very slow growth of the tumors. All experimental groups showed transient weight loss (Fig. 4D). IHC staining of the fixed tumor tissues confirmed high FAP expression in HT1080-FAP tumors (Fig. S11). Notably, obvious necrosis inside the tumors was observed in the [^177^Lu]Lu-DOTA-FD3 treatment group on hematoxylin–eosin (H&E) and IHC staining. Thus, both [^177^Lu]Lu-DOTA-FD2 and [^177^Lu]Lu-DOTA-FD3 demonstrated promising antitumor efficacy in HT1080-FAP models with [^177^Lu]Lu-DOTA-FD3 treatment resulting in a higher rate of CR.

### Radioligand therapy [^177^Lu]Lu-DOTA-FD3 in T3M-4 pancreatic cancer models

HT1080-FAP tumors have stable FAP expression on tumor cells through lentivirus transfection. In comparison, T3M-4 tumor cells are surrounded by stroma cells that naturally express FAP at a high level (Fig. S12). We then constructed T3M-4 tumor models to consolidate the therapeutic efficacy of [^177^Lu]Lu-DOTA-FD3. The models were divided into 2 groups, free ^177^Lu and 500 μCi [^177^Lu]Lu-DOTA-FD3 treatment groups (*n* = 7 per group). The tumor volume was around 135 mm^3^ right before the onset of the treatments. As shown in Fig. 4E, tumor volume in the 500 μCi [^177^Lu]Lu-DOTA-FD3 treatment group was satisfactorily controlled until the end of the observation, while the free ^177^Lu showed no treatment effect. Meanwhile, mice weight in the 500 μCi [^177^Lu]Lu-DOTA-FD3 treatment group slightly dropped than the free ^177^Lu treatment group (Fig. 4F). Thus, radioligand therapy (RLT) with [^177^Lu]Lu-DOTA-FD3 has a good therapeutic effect in stroma-rich tumors despite negative FAP expression on the tumor cells.

### Safety and biodistribution of [^68^Ga]Ga-DOTA-FD1 in healthy volunteers

**Fig. 5.**
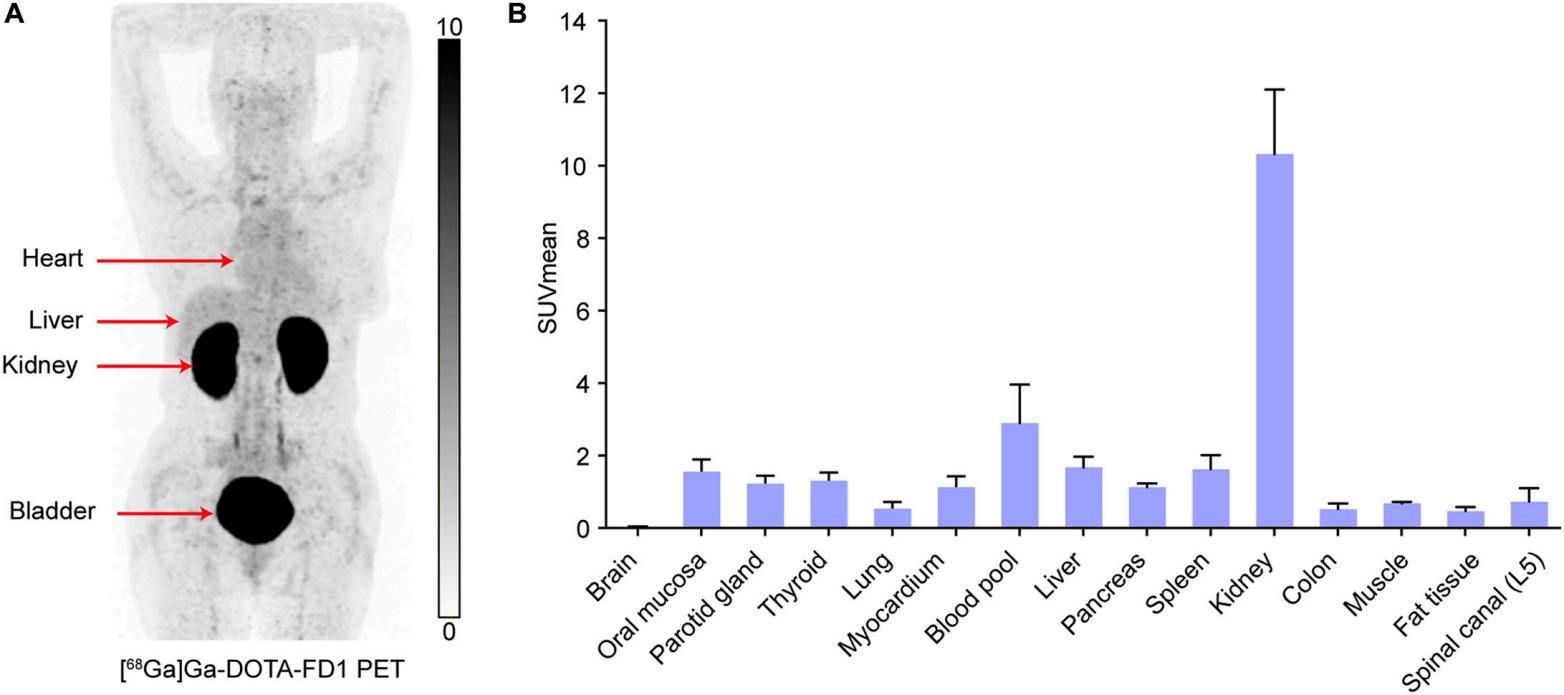
(A) Maximum projection image of [^68^Ga]Ga-DOTA-FD1 at 60 min after injection in a healthy volunteer. (B) SUVmean of normal tissues and organs (*n* = 3).

Of the reported novel FAP-targeted PET imaging and RLT radiopharmaceuticals, we first translated [^68^Ga]Ga-DOTA-FD1 for clinical use. No adverse events were observed with [^68^Ga]Ga-DOTA-FD1 in all healthy volunteers and patients during the injection and at 4 h follow-up. Figure 5 displayed a representative PET MIP image in a healthy volunteer (Fig. 5A) and biodistribution data on 3 volunteers’ normal organs (Fig. 5B). [^68^Ga]Ga-DOTA-FD1 has negligible uptake in normal tissues and organs except the kidneys, leading to a lower background. This low background can facilitate better detection of tumor lesions.

### [^68^Ga]Ga-DOTA-FD1 PET/CT imaging in tumor patients

**Fig. 6.**
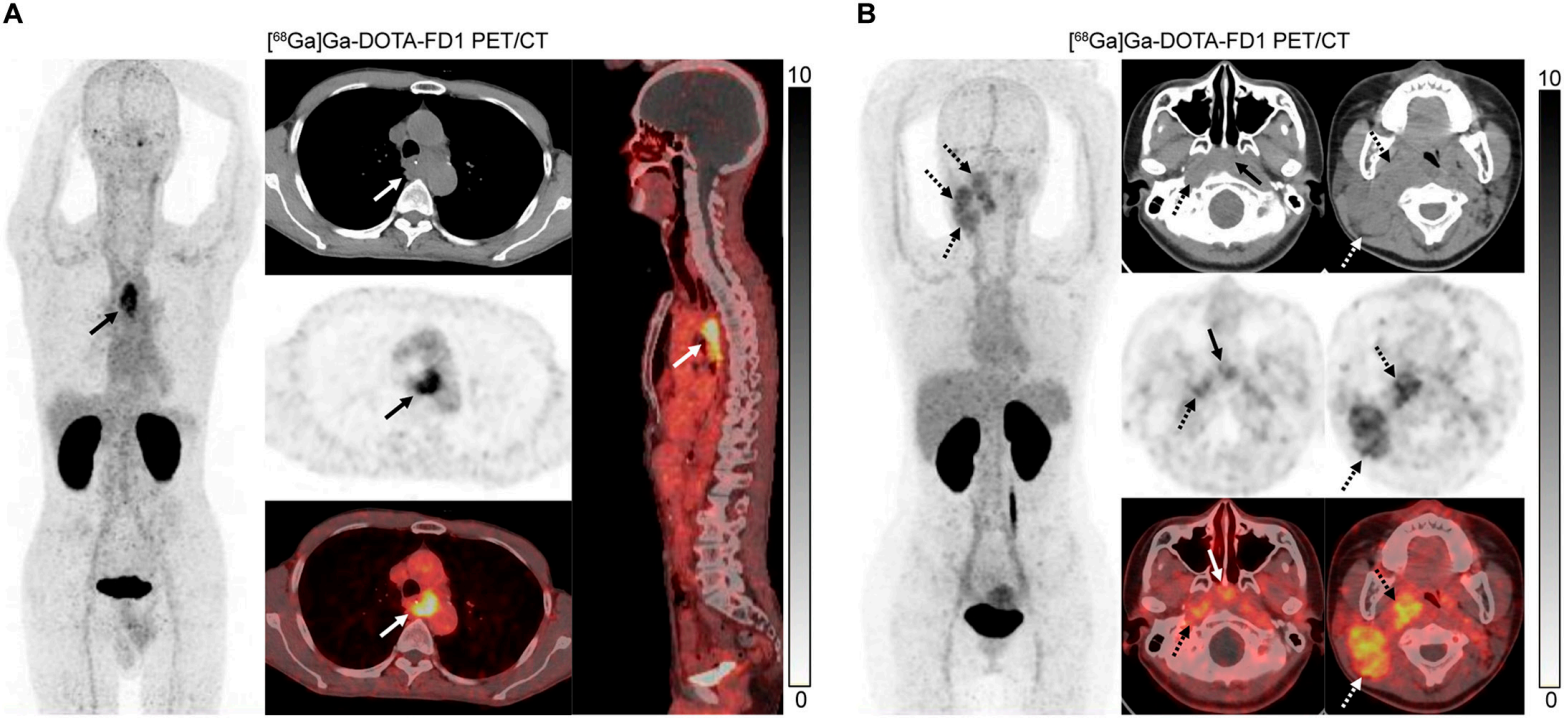
(A) An adult patient with known esophageal cancer underwent [^68^Ga]Ga-DOTA-FD1 PET/CT for initial assessment. Intense [^68^Ga]Ga-DOTA-FD1 uptake was observed in the primary lesion (black and white arrows). (B) An adult woman with known nasopharyngeal carcinoma who underwent [^68^Ga]Ga-DOTA-FD1 PET/CT for initial assessment. Intense [^68^Ga]Ga-DOTA-FD1 uptake was observed in the primary lesion (black and white arrows) and cervical lymph node metastases (black and white dotted arrows).

In this study, 6 patients (4 women, and 2 men) were enrolled for initial staging (4 patients) and recurrence detection (2 patients), including 1 patient with thyroid cancer, 2 with nasopharyngeal carcinoma (NPC), and 3 with esophageal cancer. Among the initial diagnosis stage patients, 3 individuals were diagnosed with esophageal carcinoma and 1 was diagnosed with NPC. The 4 primary lesions in these patients demonstrated high radiotracer uptakes (median SUVmax 8.86, range 5.27 to 13.42) (Fig. 6A, solid arrows). Regarding lymph node metastases, a total of 15 lymph node metastases in 3 patients showed high uptake of [^68^Ga]Ga-DOTA-FD1 (median SUVmax 5.6, range 4.02 to 7.76) (Fig. 6B, dotted arrows). For distant metastases (7 lung metastases and 1 bone metastasis), these metastases also demonstrated increased [^68^Ga]Ga-DOTA-FD1 uptake than that of the surrounding normal tissue. Detailed clinical information of the 6 patients is presented in Table.

**Table.** Summary of patient characteristics

Patient no.	Diagnosis	Clinical indication for PET	Primary tumor		Lymph node metastases		Bone and visceral metastases	
			Lesion size (cm)	Median SUVmax	Lesion size (range, cm)	Median SUVmax (range)	Lesion size (range, cm)	Median SUVmax (range)
Patient 1	Thyroid cancer	Recurrence detection	NA	NA	NA	NA	0.7 (0.43–6.27)	2.87 (2.52–4.45)
Patient 2	NPC	Recurrence detection	NA	NA	7.07 (NA)	1.17 (NA)	NA	NA
Patient 3	Esophagus cancer	Initial staging	4.71	7.64	1.03 (0.76–1.38)	4.29 (4.02–4.57)	NA	NA
Patient 4	NPC	Initial staging	1.03	5.27	1.19 (0.73–2.79)	5.67 (4.14–7.76)	NA	NA
Patient 5	Esophagus cancer	Initial staging	6.61	10.07	NA	NA	NA	NA
Patient 6	Esophagus cancer	Initial staging	5.61	13.42	NA	NA	NA	NA

NPC, nasopharyngeal carcinoma; NA, not applicable.

## Discussion

Targeted therapy is one of the most researched treatments under current investigation for malignant tumors, which means the cell-killing drugs specifically target the surface antigen of tumor cells resulting in the tumor cells’ death [35,36]. Another branch of target-based therapy is targeted radionuclide therapy (TRT), in which the therapeutic drug contains not only a unit binding to antigens on the surface of tumor cells but also a therapeutic radionuclide such as ^177^Lu or ^90^Y that delivers ionization energy [37]. The Food and Drug Administration approved ^177^Lu-DOTATATE in 2018 for treating gastroenteropancreatic neuroendocrine tumors in patients with somatostatin receptor positivity [38]. In the meantime, FAP is present in a broad range of tumors and its variability is linked to the effectiveness of tumor therapies [39]. Herein, FAP-targeted RLT is a promising strategy for FAP-positive or stroma-rich tumors, such as sarcoma directly expressing FAP on the tumor cells or pancreatic cancers with abundant stroma. In recent years, FAPIs including FAPI-04 and FAPI-46 presented encouraging results in tumor imaging and attracted numerous studies focusing on efficacy research [19,40,41]. Many clinical studies have reported the antitumor efficacy of ^177^Lu- or ^90^Y-labeled FAPIs [25,42]. However, because of the comparatively short tumor retention of FAPI-04 and FAPI-46, the antitumor efficacy was not persistent and significant. The lack of desired results made researchers develop many strategies to prolong the circulation time of FAPIs to increase tumor retention. Several FAPI derivatives were produced and the diagnostic or theranostic potentials were explored in preclinical models [24].

In our work, we produced 3 peptides named FD1, FD2, and FD3, which contain the chelator DOTA for labeling with ^68^Ga and ^177^Lu for diagnosis and theranostics, respectively. All the 3 peptides could bind tightly to recombinant human and murine FAP proteins. [^68^Ga]Ga-DOTA-FD1 could sharply and specifically delineate the subcutaneous HT1080-FAP xenografts with FAP overexpression. More importantly, the clinical translation of [^68^Ga]Ga-DOTA-FD1 confirmed the safety profiles and diagnostic value of the tracer in various cancer types including thyroid cancer, NPC, and esophagus cancer. The uptake of [^68^Ga]Ga-DOTA-FD1 in organs other than the kidneys was low, leading to satisfactory tumor-to-background contrast. [^68^Ga]Ga-DOTA-FD1 not only detected primary tumor lesions but also showed intense uptake in lymph node metastases and distant metastases (e.g., lungs and bone metastases). Renal accumulation of [^68^Ga]Ga-DOTA-FD1 remained at a high level for unknown reasons and we are optimizing the pharmacokinetics and pharmacodynamics by adapting new linkers and conjugation strategies. Moreover, we will thoroughly evaluate the diagnostic performance of these tracers by adapting the state-of-art total-body dynamic PET/CT scanner, which allows real-time visualization of the pharmacokinetics of radiotracers [43].

The short in vivo circulation time of FD1 does not allow for effective TRT. Several strategies to prolong circulation time and increase tumor retention of FAPIs have been investigated, including the incorporation of butyric acid conjugation, truncated Evans Blue, and dimerization [44]. We adapted 4-(p-iodophenyl) butyric acid moiety to modify FD1 to improve the circulation and distribution profiles for FAP-targeted treatment. Both FD2 and FD3 derived from FD1 have excellent binding affinities both to human and murine FAP, warranting fair and accurate evaluation of the therapeutic value in murine models. We then developed 2 therapeutic radiopharmaceuticals ([^177^Lu]Lu-DOTA-FD2 and [^177^Lu]Lu-DOTA-FD3) and thoroughly explored the antitumor efficacies in preclinical models. At 45 h after injection of [^177^Lu]Lu-DOTA-FD3, the highest radioactivity accumulation was found in the blood pool, whereas at 90 h, the highest radioactivity uptake was already found in the tumor which was supposed to increase in a time-dependent manner. The relatively long circulation of radiopharmaceuticals in the blood pool brings the advantage of higher tumor uptake/more substantial therapeutic efficacy on the one hand and the inevitable degree of hematotoxicity on the other hand. In our preclinical findings, treatment with 400 to 600 μCi of [^177^Lu]Lu-DOTA-FD3 substantially suppressed the growth of the tumors within the first 2 weeks followed by rebounding of tumor volume, resulting in temporary but not sustained tumor remission. A preclinical study showed that multi-cycle low-dose administration of ^177^Lu-labeled FAPI of long circulation time showed a good tumor control rate within 2 months [26]. We are currently testing whether a second administration of low-dose [^177^Lu]Lu-DOTA-FD3 at day 15 can control tumor progression. Meanwhile, we found that a single administration of 200 to 500 μCi of [^177^Lu]Lu-DOTA-FD3 satisfactorily suppressed the growth of tumors of small volumes at the onset of the therapies, producing durable responses in both HT1080-FAP and T3M-4 tumor models at the endpoint of efficacy monitoring. This result provides a thought that [^177^Lu]Lu-DOTA-FD2 or [^177^Lu]Lu-DOTA-FD3 TRT may be a viable option for patients with small primary lesions or micrometastases. Our initial data showed that [^177^Lu]Lu-DOTA-FD3 may be advantageous over [^177^Lu]Lu-DOTA-FD2 in cancer therapy; however, further research is required to reinforce this assertion. The long circulation time of FD2/FD3 will inevitably cause hematological toxicity while ensuring therapeutic efficacy [45]. Keane et al. [46] demonstrated that the circulating FAP activity level varies between species, and the circulating FAP activity in mice is 15 to 20 times higher than that in primates, so the hematological toxicity induced by FAP-targeted RLT in humans may be lower than the hematological toxicity detected in mice, which will be focused on in our subsequent clinical trials in humans. In addition, all mice in the treatment group had some degree of body weight loss, possibly related to the depletion of FAP+ stromal cells. Depletion of FAP+ stromal cells causes cachexia, leading to a loss of muscle mass despite adequate food intake [45].

Based on the available data, we can conclude that [^68^Ga]Ga-DOTA-FD1 and [^177^Lu]Lu-DOTA-FD2/[^177^Lu]Lu-DOTA-FD3 are promising theranostic pairs for managing FAP-overexpressing tumors. The presence of FAP-expressing CAFs is crucial in tumorigenesis and development, and their depletion is associated with tumor shrinkage [47–49]. Several studies have shown that depletion of FAP-expressing stromal cells overcomes immunosuppression in non-immunodeficient normal mice, eliciting the immune system and a number of cytokines that promote rapid tumor and stromal cell death and allow for immune control of growth [47,50,51]. Follow-up is needed to further validate the therapeutic efficacy of FAP-targeted radioligands in preclinical models of FAP transgenic mice, as well as the associated toxicities. In most solid tumors, the mechanism of action of TRT targeting FAP is to damage CAFs in the stroma surrounding tumor cells, as well as cross-fire effect to kill some tumor cells, but does not have a direct targeted killing effect on all tumor cells [23]. However, it should be noted that for some specific cancer type, FAP was highly expressed on both the tumor cells and stroma (e.g., sarcoma and mesothelioma). In these specific tumor types, FAP-specific TRT can directly kill the tumor cells. Disease progression was frequently observed when using monotherapy, necessitating a change in therapeutic strategy. Therefore, the combination of some other treatments (e.g., immunotherapy, chemotherapy, and targeted therapy) may lead to a more satisfactory and long-lasting therapeutic effect. Our laboratory is actively exploring the synergistic therapeutic efficacy of FAP TRT with antibody–drug conjugate in stromal-abundant malignant tumors.

In the work, we have successfully developed a series of ^68^Ga/^177^Lu-labeled peptide radiopharmaceuticals for FAP-targeted theranostics. Of them, [^68^Ga]Ga-DOTA-FD1 has good diagnostic value in various solid tumors with the diagnostic value confirmed in a pilot clinical trial. [^177^Lu]Lu-DOTA-FD2 and [^177^Lu]Lu-DOTA-FD3 demonstrate confirmative treatment efficacies in preclinical solid tumor models. Further optimization and application of these novel theranostic agents will enrich the toolbox for managing FAP-positive tumors.

## Materials and Methods

### Binding affinity measurement

The Biacore SPR interaction study was conducted to determine the binding kinetics between the peptides and the immobilized human FAP protein (FAP-H5244, ACRO Biosystems) or murine FAP protein (FAP-M52H3, ACRO Biosystems) as target antigens. The final binding affinity, represented by the equilibrium dissociation degree (M) or *K*_D_, was calculated by examining the relationship between the binding rate constant (*k*_a_) (M^−1^ s^−1^) and the dissociation rate (*k*_d_) (s^−1^).

### Radiolabeling and quality control

To perform ^68^Ga-labeling, approximately 20 nmol of FD1, FD2, or FD3 was dissolved in 20 μl of dimethyl sulfoxide and 230 μl of sterile deionized water. This mixture was added to 400 μl of ^68^GaCl_3_ solution (containing 37 MBq) in 0.1 M HCl, with pH adjusted to 4.0 using 0.1 M NH_4_OA_C_. The mixture was incubated at 100 °C for 10 min without any additional purification steps. For ^177^Lu-labeling, high-purity radionuclide ^177^LuCl_3_ in 0.04 M HCl (3.9 GBq in 97 μl) was purchased from ITM (Munich, Germany). ^177^LuCl_3_ solution (555 MBq in 0.04 M HCl) was added to 500 μl of 1M NH_4_OA_C_ (pH = 5), followed by the addition of 375 nmol of FD1, FD2, or FD3. The solution was shaken at a temperature of 100 °C for a duration of 30 min. Instant thin-layer chromatography (iTLC; Eckert & Ziegler Radiopharma Inc.) was used to evaluate the RCP of the final products. For iTLC, the mobile phase consisted of 1 M NH_4_OA_C_ and methyl alcohol in a ratio of 1:1. The final solution must be adjusted to pH 7.0 before being injected into the experimental mice via the tail vein. The stability of ^177^Lu-labeled radiopharmaceuticals was evaluated in a mixture of 50% FBS and 50% PBS at 2 h and 24 h after preparation. In the process of preparing each [^68^Ga]Ga-DOTA-FD1 for clinical imaging, the end product underwent sterilization by passing it through a 0.22-μm Millipore filter.

### Preparation of tumor models

A human pancreatic cancer cell line BxPC-3 and a murine pancreatic cancer cell Panc-02 were obtained from the American Type Culture Collection. The pancreatic cancer cell line T3M-4 was purchased by MeisenCTCC (Zhejiang, China). BxPC-3 and T3M-4 cell lines were grown in RPMI 1640 medium (Shanghai BasalMedia Technologies Co., LTD.) containing 10% FBS (GE Healthcare, Chicago, IL, USA) and 1% penicillin–streptomycin solution (Invitrogen). To culture the Panc-02 cell line, a medium called Dulbecco’s modified Eagle medium from Shanghai BasalMedia Technologies Co., LTD. was used, which consisted of 10% FBS. The cell line HT-1080 was grown in MEM medium (Shanghai BasalMedia Technologies Co., LTD.) supplemented with 10% FBS, 1% penicillin-streptomycin solution, and 1% nonessential amino acids (NEAA-11140050; Gibco). A human fibrosarcoma cell line was stably transfected with FAP (HT1080-FAP) and cultured, as we previously described [52]. All cell lines were cultured in a humid incubator with 5% CO_2_ at a temperature of 37 °C. Approval was granted by the Institutional Animal Care and Use Committee (Renji Hospital, School of Medicine, Shanghai Jiao Tong University) for all experimental protocols and procedures related to animal care. Panc-02 cells were implanted by subcutaneous injection of 5 × 10^6^ tumor cells suspended in 100 μl PBS and matrigel matrix (Corning) with equal volume into the upper limb flank of C57BL/6 mice (Shanghai Sheng Chang Biotechnology Co., Ltd.). Four- to 6-week-old thymus-free nude mice (GemPharmatech) were subcutaneously inoculated with BxPC-3, T3M-4, HT-1080, and HT1080-FAP cells (5 × 10^6^ in 100 μl of PBS mixing with 100 μl matrigel matrix) in the right shoulder. Follow-up microPET/CT imaging and ex vivo biodistribution experiments were performed when the tumor diameter reached 8 to 9 mm.

### MicroPET imaging study

MicroPET imaging was performed on isoflurane-anesthetized mice injected intravenously via the lateral tail vein with around 3 MBq (2.5 nmol) of the radioligand at certain times. Regarding the FD1 blocking group, an excessive amount of unlabeled FD1 (200-fold) was simultaneously injected with [^68^Ga]Ga-DOTA-FD1 into the tail vein of another batch of mice. Images were acquired using an IRIS PET/CT system (Inviscan Imaging Systems). PET data were reconstructed using a nonscatter-corrected 3D-ordered subset expectation optimization/maximum a posteriori (OSEM3D/MAP) algorithm. The information was analyzed using OsiriX Lite software (Pixmeo SARL) and Inveon Research Workplace (Siemens Preclinical Solutions). The percentage of injected dose per gram of tissue (%ID/g, mean ± SD) was utilized to present the distribution of tracers in the ROI, encompassing tumor, heart, lung, liver, kidney, and muscle. The detailed values are shown in Table S1.

### TRT with [^177^Lu]Lu-DOTA-FD1/FD2/FD3 in FAP-overexpressing models

HT1080-FAP tumor models with higher levels of FAP expression were utilized in this study. Tumor-bearing mice were subjected to therapy experiments when the tumor volume reached 50 to 180 mm^3^. Tumor models with different tumor volumes were injected with different therapeutic doses. Mice bearing HT1080-FAP tumors of slightly larger tumor volumes were randomly assigned into 4 groups (*n* = 5 per group): control group and treatment groups with 800 μCi of [^177^Lu]Lu-DOTA-FD1, 400 μCi of [^177^Lu]Lu-DOTA-FD3, and 600 μCi [^177^Lu]Lu-DOTA-FD3. Mice bearing HT1080-FAP tumors of slightly smaller tumor volumes were also divided into 4 groups (*n* = 6 per group): control group and treatment groups with 200 μCi of [^177^Lu]Lu-DOTA-FD1, 200 μCi of [^177^Lu]Lu-DOTA-FD2, and 200 μCi of [^177^Lu]Lu-DOTA-FD3. Tumor volume and mice body weight were examined daily after a single-dose injection. Weight monitoring is expressed as a percentage of initial weight on the measurement day. Tumor volume was measured as half of the length × width squared times. Mice were euthanized when the tumor volume exceeded 1,500 mm^3^, the tumor ruptured, or when it lost more than 20% of its body weight. T3M-4 tumor models with abundant stroma were established to validate the therapeutic efficacy of [^177^Lu]Lu-DOTA-FD3. When the tumor volume grew to 100 to 150 mm^3^, the mice were randomly divided into 2 groups (*n* = 7 per group): free ^177^Lu treatment group and 500 μCi [^177^Lu]Lu-DOTA-FD3 treatment group. Tumor volume and mice body weight were examined every 2 days. The detailed dose of each group was summarized in Tables S2 to S4.

### Ex vivo biodistribution and histopathological staining studies

After imaging collection and image reconstruction, samples from euthanized experimental mice, including blood and multiple significant organs, were harvested and weighed. The radioactivity in each collected tissue was counted using an automated gamma counter (Wizard 2480, PerkinElmer) and the accumulation of radioactivity in different tissues and organs was quantified and expressed as %ID/g (mean ± SD). [^177^Lu]Lu-DOTA-FD1 (~1.11 MBq, *n* = 3), [^177^Lu]Lu-DOTA-FD2 (~1.11 MBq, *n* = 3), and [^177^Lu]Lu-DOTA-FD3 (~1.11 MBq, *n* = 3) were injected into the tail vein of free nude mice at 6 h, 24 h, and 24 h for ex vivo biodistribution to verify the circulation time of the 3 probes in vivo. Biodistribution data from tumor-bearing mice injected with [^177^Lu]Lu-DOTA-FD3 at 45 h (~1.11 MBq, *n* = 4) and 90 h (~1.11 MBq, *n* = 3) validated the prolonged enrichment of the probe at the tumor site. At the end of each probe imaging and radioactivity counting study, tumor tissues were fixed in paraformaldehyde tissue fixative. Briefly, sections of 10 μm were cut and stained for H&E following the standard protocols. IHC staining with a FAP antibody (ab227703, Abcam) was performed only on tumor tissues following accepted techniques.

### PET/CT imaging in healthy volunteers and patients with tumor

This clinical trial received ethical approval from the institutional review board of The First Affiliated Hospital of Xiamen University and has been registered at ClinicalTrials.gov (NCT04849247). All healthy volunteers (*n* = 3) and patients (*n* = 6) provided written informed consent before participation. There was no need for any particular preparation for [^68^Ga]Ga-DOTA-FD1 PET/CT. Each participant was administered a dose of [^68^Ga]Ga-DOTA-FD1 at a rate of 3.0 to 3.7 MBq/kg. Adverse events were under observation for 4 h after the injection of [^68^Ga]Ga-DOTA-FD1. A hybrid PET/CT system (Discovery MI, GE Healthcare, Milwaukee, WI, USA) was used to perform static PET/CT imaging after 60 min of injection. PET/CT scan was conducted from the head region to the upper thighs (for [^68^Ga]Ga-DOTA-FD1 PET/CT). For the CT scan, the parameters utilized were 110 kV, 80 mA, and a slice thickness measuring 3.75 mm. The obtained data were transferred to the Advantage Workstation (version AW 4.7, GE Healthcare). The Bayesian penalized likelihood reconstruction algorithm (Q.clear, GE Healthcare) was utilized for image reconstruction, employing a penalization factor (beta) of 500. For tumor staging/restaging, a total of 6 patients received paired PET/CT imaging using [^68^Ga]Ga-DOTA-FD1. In terms of quantitative assessment, the maximum standardized uptake value (SUVmax) and the mean SUV (SUVmean) were employed to quantify the uptake of the radiopharmaceutical in both normal organs and tumor tissues.

### Statistical analysis

Prism software (Version 9.0, GraphPad Software) and SPSS 22.0 software (IBM, USA) were utilized for conducting the statistical analyses. Means were compared using both a 2-way analysis of variance and a Student’s *t* test. A significance level of less than 0.05 was deemed as statistically significant. For the radiotracer uptakes of normal organs, the mean value accompanied by the standard deviation was used. Regarding the radiotracer uptakes within tumor lesions, the depiction employed was the median value along with the range.

### Study approval

The experiment was approved by the institutional clinical research ethics committee of Renji Hospital, School of Medicine, Shanghai Jiao Tong University. All experimental protocols and animal care procedures were approved by the Institutional Animal Care and Use Committee (Renji Hospital, School of Medicine, Shanghai Jiao Tong University). This clinical trial received ethical approval from the institutional review board of The First Affiliated Hospital of Xiamen University.

## Data Availability

The data used to support the findings of this study are available from the corresponding author upon request.
