# Supplementary material for: Development and Characterization of Novel FAP-Targeted Theranostic Pairs: A Bench-to-Bedside Study
Source: Research (Wash D C). 2023 Nov 28;6:0282. doi: 10.34133/research.0282 (PMC11066877; doi:10.34133/research.0282)
Supplement: Supplementary 1 — Figs. S1 to S12 Table S1 [file research.0282.f1.docx]

**Development and Characterization of Novel FAP-targeted Theranostic Pairs: A Bench to Bedside Study**

Wei Huang^1, #^, Yizhen Pang^2, #^, Qiufang Liu^3^, Chenyi Liang^1^, Shuxian An^1^, Qianyun Wu^1^, You Zhang^1^, Gang Huang^1^, Haojun Chen^2,*^, Jianjun Liu^1, *^, Weijun Wei^1,*^

^1^Department of Nuclear Medicine, Institute of Clinical Nuclear Medicine, Renji Hospital, School of Medicine, Shanghai Jiao Tong University, Shanghai 200127, China.

^2^Department of Nuclear Medicine and Minnan PET Center, Xiamen Cancer Center, Xiamen Key Laboratory of Radiation Oncology, The First Affiliated Hospital of Xiamen University, School of Medicine, Xiamen University, Xiamen 361003, China.

^3^Department of Nuclear Medicine, Fudan University Shanghai Cancer Center, Fudan University, Shanghai 200032, China.

^#^ The authors contributed equally to the work.

**^*^ Corresponding Authors:**

**Prof. Haojun Chen**

Department of Nuclear Medicine and Minnan PET Center, The First Affiliated Hospital of Xiamen University, School of Medicine, Xiamen University, Xiamen 361003, China; e-mail: [leochen0821@foxmail.com](mailto:leochen0821@foxmail.com).

**Prof. Jianjun Liu**

Department of Nuclear Medicine, Renji Hospital, School of Medicine, Shanghai Jiao Tong University, Shanghai 200127, China; e-mail: [nuclearj@163.com](mailto:nuclearj@163.com).

**Prof. Weijun Wei**

Department of Nuclear Medicine, Renji Hospital, School of Medicine, Shanghai Jiao Tong University, Shanghai 200127, China; e-mail: [wwei@shsmu.edu.cn](mailto:wwei@shsmu.edu.cn).


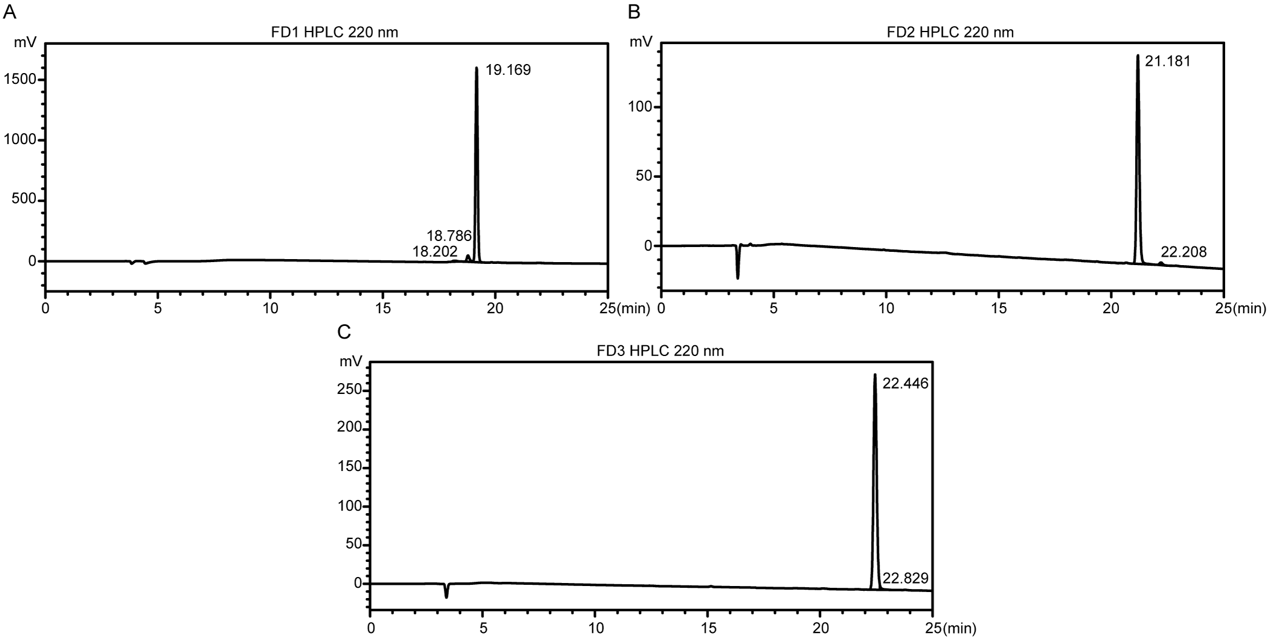


**Figure S1.** The purity of FD1/FD2/FD3 was assessed by high performance liquid chromatography (HPLC). The purity of was 95% for FD1 (**A**), 98% for FD2 (**B**), and 99% for FD3(**C**).


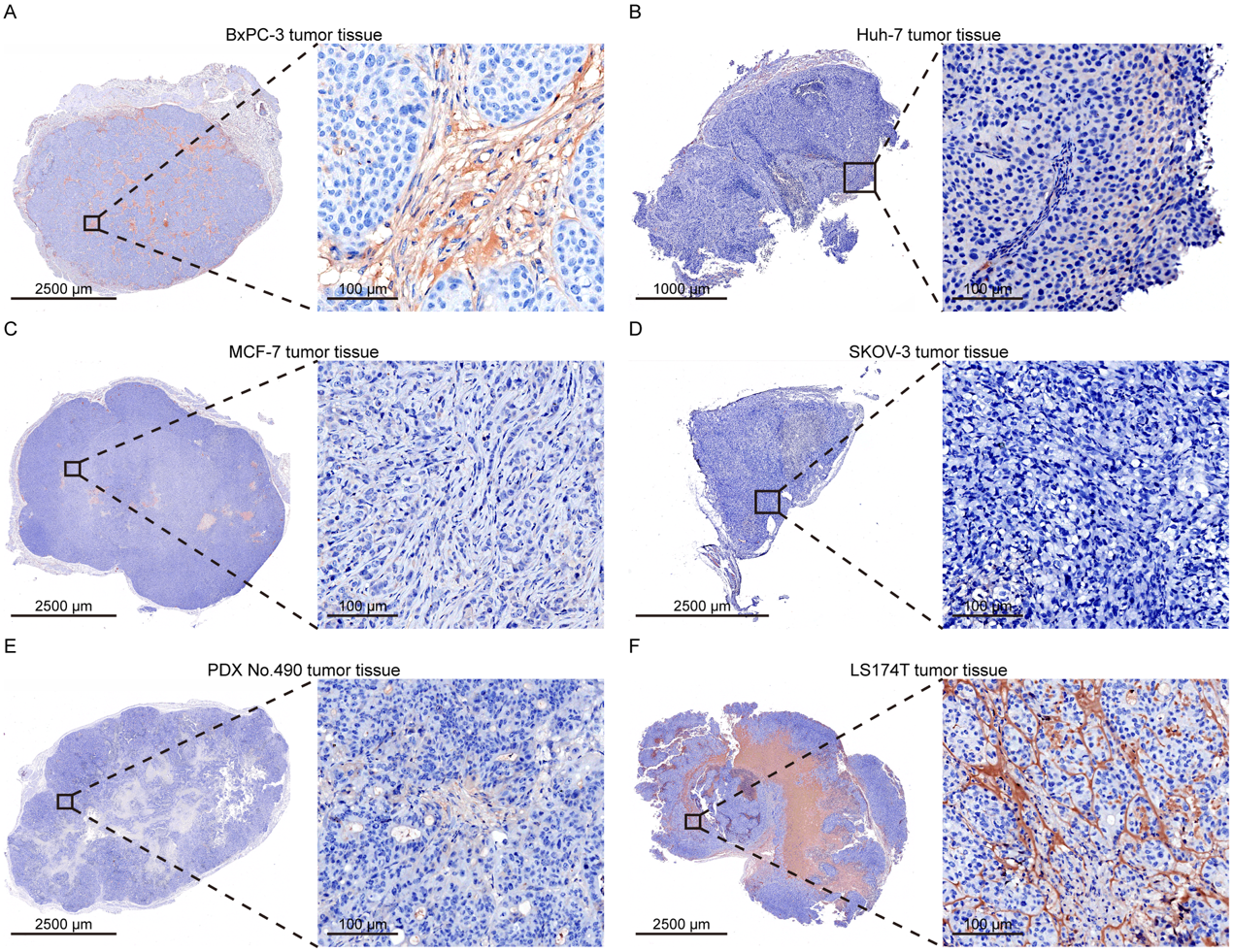


**Figure S2.** Immunohistochemical (IHC) staining results of the different types of tumor tissues used FAP protein. (**A**) BxPC-3 pancreatic adenocarcinoma tissue. (**B**) Huh-7 hepatocellular carcinoma tissue. (**C**) MCF-7 breast cancer tissue. (**D**) SKOV-3 ovarian cancer tissue. (**E**) No.490 patient-derived gastric cancer xenograft. (**F**) LS174T colorectal adenocarcinoma tissue.


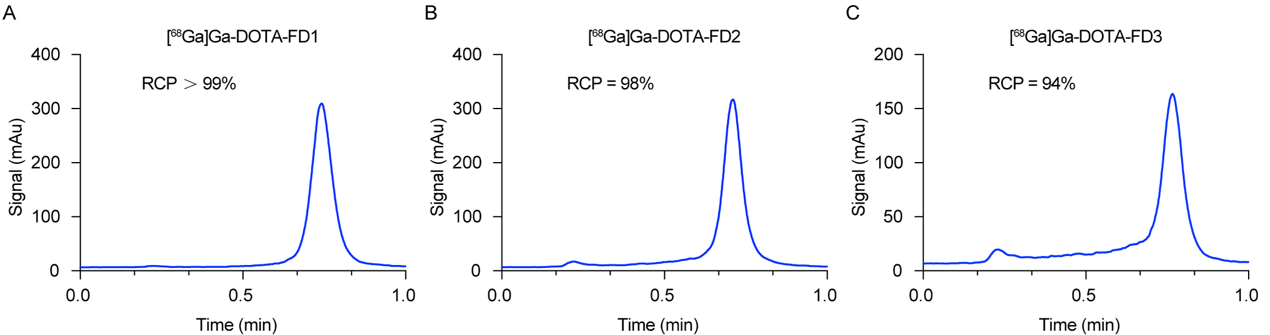


**Figure S3.** Radiochemical purity (RCP) of ^68^Ga-labeled probes ([^68^Ga]Ga-DOTA-FD1, [^68^Ga]Ga-DOTA-FD2, and [^68^Ga]Ga-DOTA-FD3). (**A**) RCP of [^68^Ga]Ga-DOTA-FD1 was >99%. (**B**) RCP of [^68^Ga]Ga-DOTA-FD2 was 98%. (**C**) RCP of [^68^Ga]Ga-DOTA-FD3 was 94%.


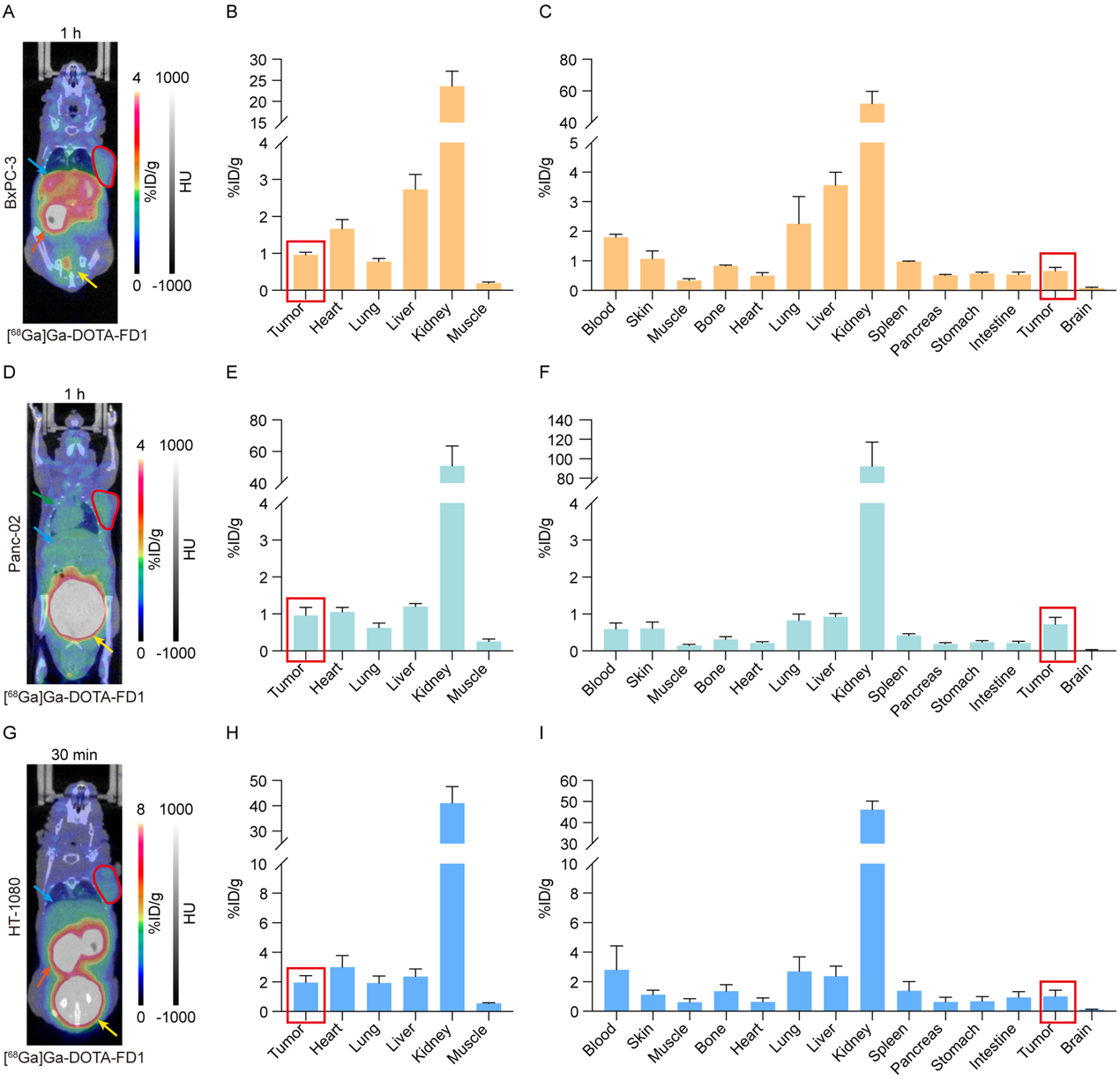


**Figure S4.** [^68^Ga]Ga-DOTA-FD1 immunoPET imaging in kinds of subcutaneous tumor models. (**A**, **D**, and **G**) [^68^Ga]Ga-DOTA-FD1 immunoPET imaging in BxPC-3 pancreatic adenocarcinoma (**A**, *n* = 3) at 1 h p.i., Panc-02 mouse pancreatic cancer (**D**, *n* = 4) at 1 h p.i., and HT-1080 fibrosarcoma (**G**, *n* = 4) models at 30 min p.i.. PET/CT images showed no significant uptake in xenografts. Tumor: red cycles. Liver: blue arrows. Kidney: orange arrows. Bladder: yellow arrows. (**B**, **E**, and **H**) The ROI analysis results of BxPC-3 (**B**, *n* = 3), Panc-02 (**E**, *n* = 4), and HT-1080 (**H**, *n* = 4) tumor models. (**C**, **F**, and **I**) The *ex vivo* distribution of [^68^Ga]Ga-DOTA-FD1 in tumors and the major organs of BxPC-3 (**C**, *n* = 3), Panc-02 (**F**, *n* = 4), and HT-1080 (**I**, *n* = 3) tumor models.


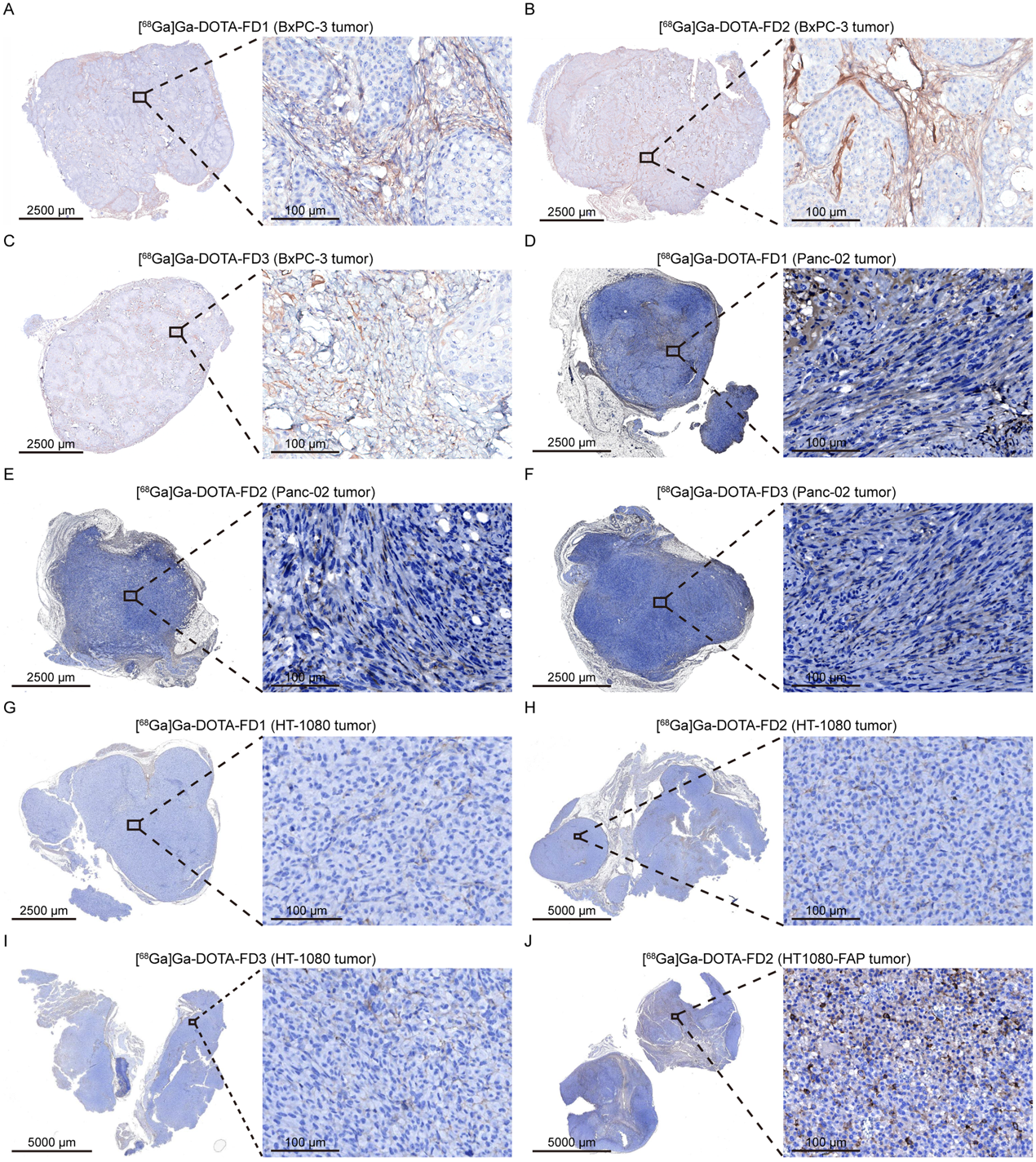


**Figure S5.** IHC staining of the resected tumors after imaging. (**A–I**) The FAP antigen immunohistochemical staining of the fixed BxPC-3 (**A–C**), Panc-02 (**D–F**), and HT1080 (**G–I**) tumors after [^68^Ga]Ga-DOTA-FD1/FD2/FD3 imaging. (**J**) IHC staining of the HT1080-FAP tumors after [^68^Ga]Ga-DOTA-FD2 imaging.


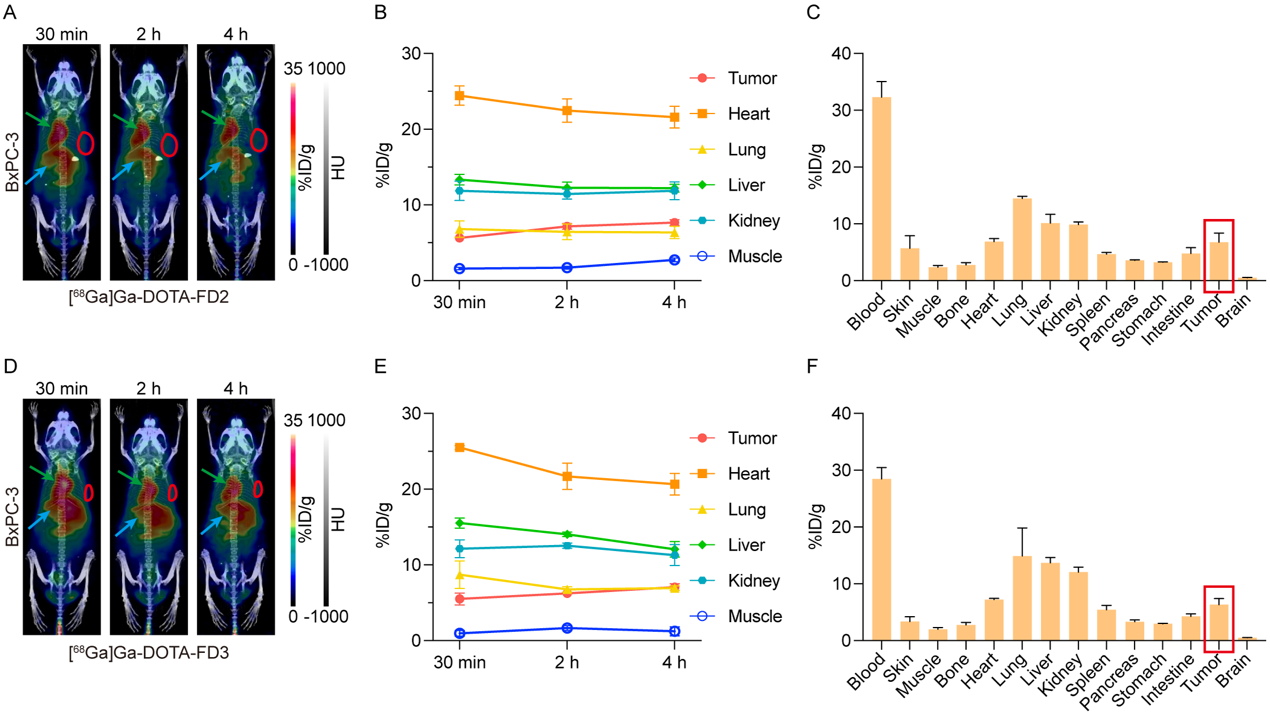


**Figure S6.** [^68^Ga]Ga-DOTA-FD2 and [^68^Ga]Ga-DOTA-FD3 immunoPET imaging in BxPC-3 tumor models at several time points. (**A**, **D**) Coronal PET/CT images of [^68^Ga]Ga-DOTA-FD2 (**A**, *n* = 3) and [^68^Ga]Ga-DOTA-FD3 (**D**, *n* = 3) imaging in BxPC-3 tumor models at 30 min, 2 h, and 4 h post-injection. The probes gradually decreased in circulation (green arrows) while tumor (red cycles) uptake gradually increased. Blue arrows indicated the Liver. (**B**, **E**) The ROI data showed the dynamic change of radioactive uptake in the tumors and major organs over time. (**C**, **F**) The *ex vivo* distribution of [^68^Ga]Ga-DOTA-FD2 (**C**, *n* = 3) and [^68^Ga]Ga-DOTA-FD3 (**F**, *n* = 3) in tumors and the major organs of BxPC-3 models after 4 h imaging finished.


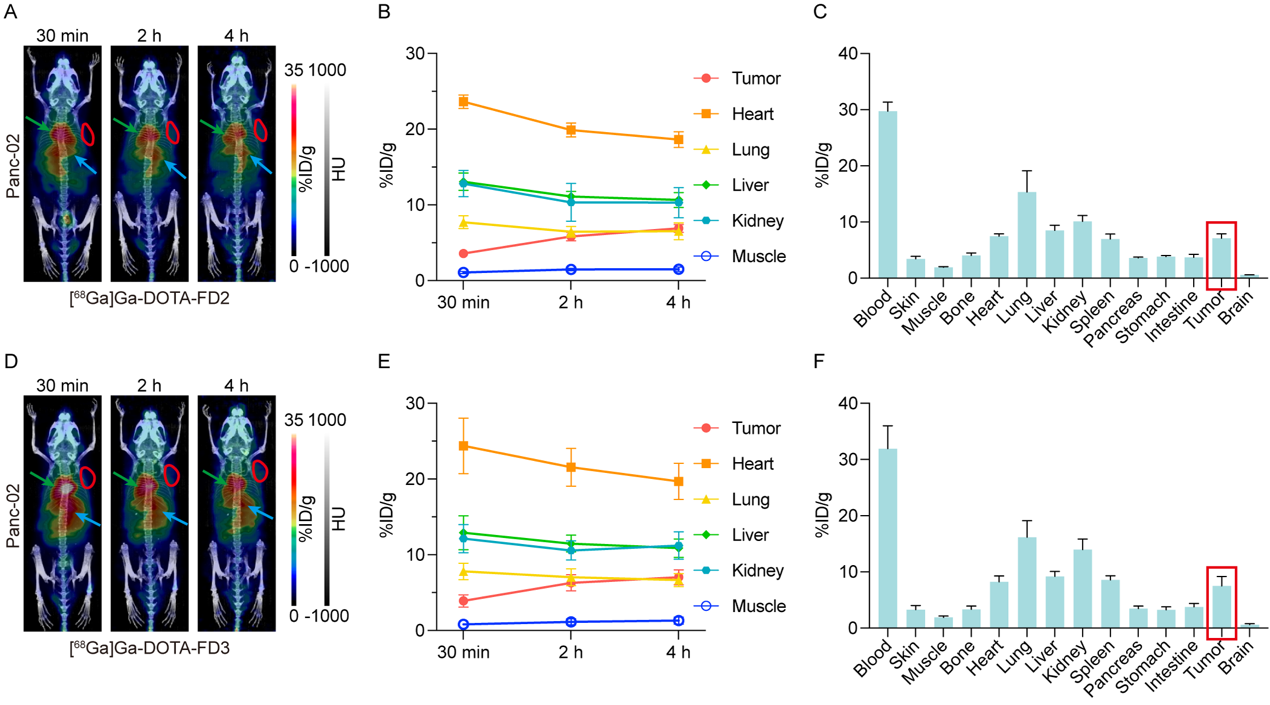


**Figure S7.** [^68^Ga]Ga-DOTA-FD2 and [^68^Ga]Ga-DOTA-FD3 PET/CT imaging in Panc-02 cell-derived xenografts (CDX) models at several timepoints. (**A**, **D**) [^68^Ga]Ga-DOTA-FD2 (**A**, *n* = 4) and [^68^Ga]Ga-DOTA-FD3 (**D**, *n* = 4) imaging in Panc-02 CDX models at 30 min, 2 h, and 4 h post-injection. Coronal images showed intensive accumulation in the heart (green arrows). Tumor: red cycles. Liver: blue arrows. (**B**, **E**) ROI analysis data showing the kinetics of [^68^Ga]Ga-DOTA-FD2 (**B**, *n* = 4) and [^68^Ga]Ga-DOTA-FD3 (**E**, *n* = 4) at different time points (30 min, 2 h, and 4 h). (**C**, **F**) The radioactive biodistribution results showed the accumulation of [^68^Ga]Ga-DOTA-FD2 (**C**, *n* = 4) and [^68^Ga]Ga-DOTA-FD3 (**F**, *n* = 4) in Panc-02 models.


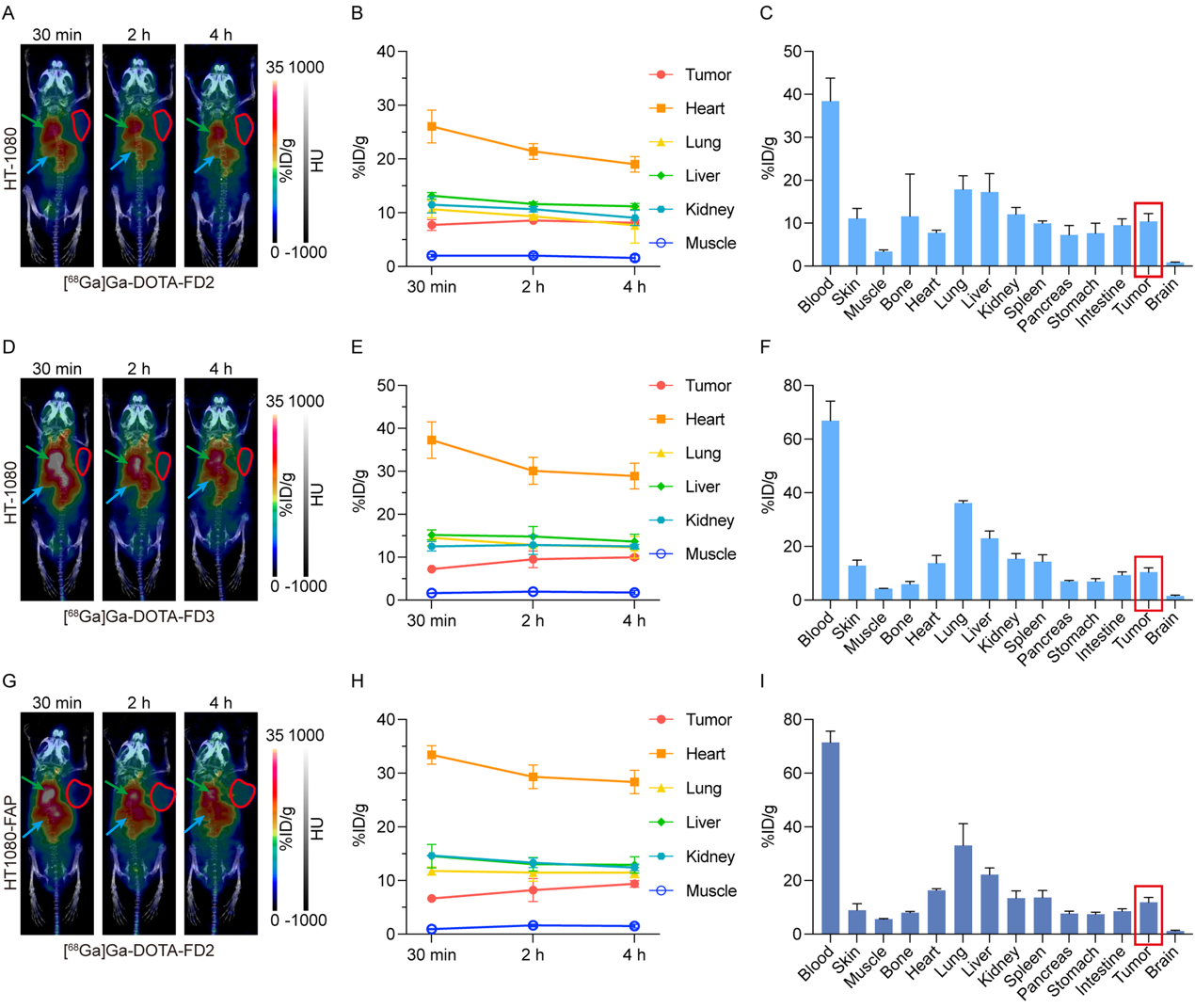


**Figure S8.** [^68^Ga]Ga-DOTA-FD2 and [^68^Ga]Ga-DOTA-FD3 PET/CT imaging in HT-1080 and HT1080-FAP CDX models at several timepoints. (**A**, **D**, and **G**) Representative static PET imaging of [^68^Ga]Ga-DOTA-FD2/FD3 in HT-1080 models (**A**, **D**, *n* = 3/group) and [^68^Ga]Ga-DOTA-FD2 in HT1080-FAP models (**G**, *n* = 3). (**B**, **E**, and **H**) The dynamic time-radioactivity curves of [^68^Ga]Ga-DOTA-FD2 and [^68^Ga]Ga-DOTA-FD3 in tumor, heart, lung, liver, kidney, and muscle of HT-1080 (**B**, **E**, *n* = 3/group) and HT1080-FAP (**H**, *n* = 3) CDX models. (**C**, **F**, and **I**) The radioactive biodistribution of [^68^Ga]Ga-DOTA-FD2 and [^68^Ga]Ga-DOTA-FD3 in HT-1080 (**C**, **F**) and [^68^Ga]Ga-DOTA-FD3 in HT1080-FAP (**I**) models, 4 h after injection (*n* = 3/group).


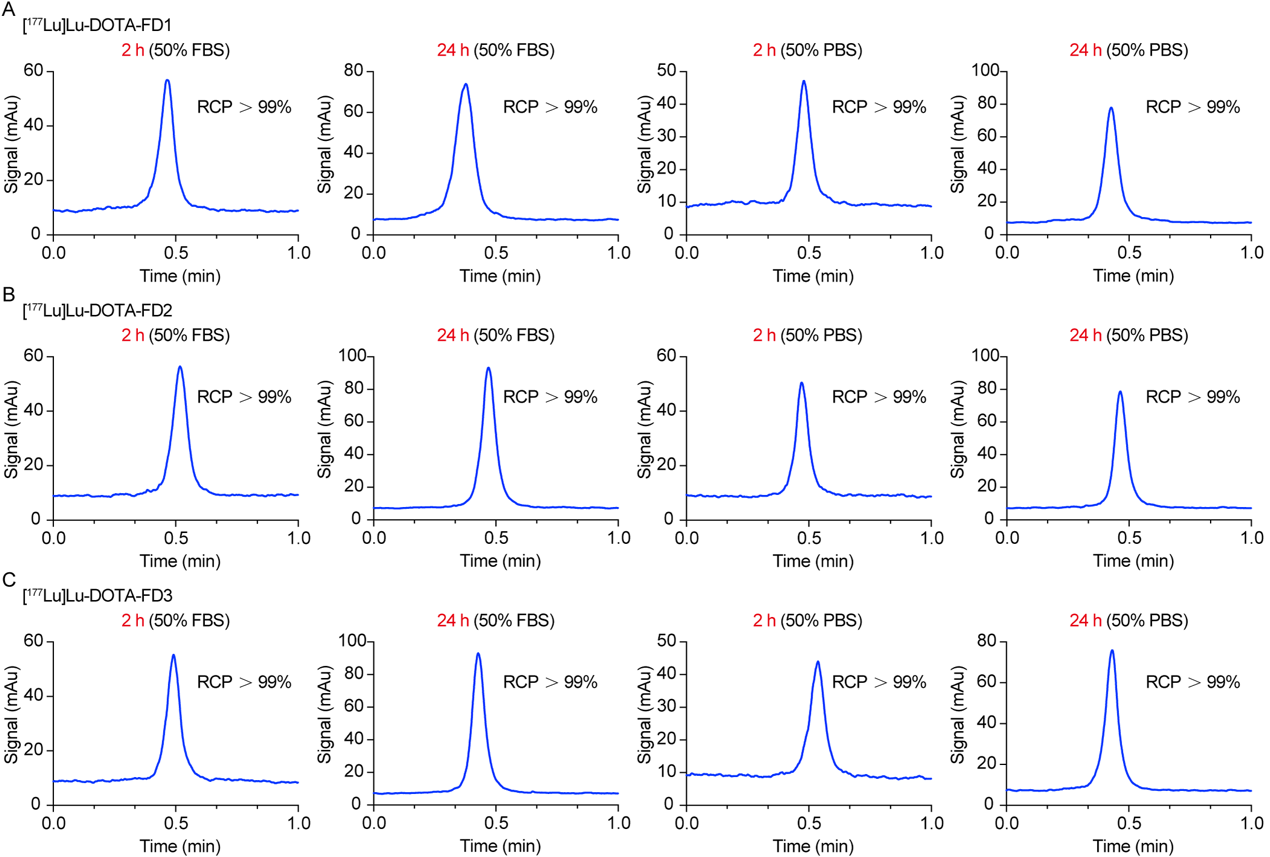


**Figure S9.** Stability of [^177^Lu]Lu-DOTA-FD1/FD2/FD3 in 50% phosphate-buffered saline (PBS) and 50% fetal bovine serum (FBS) at 2 h and 24 h. (**A–C**) [^177^Lu]Lu-DOTA-FD1 (**A**), [^177^Lu]Lu-DOTA-FD2 (**B**), and [^177^Lu]Lu-DOTA-FD3 (**C**) in 50% PBS and 50% FBS at 2 h and 24 h.


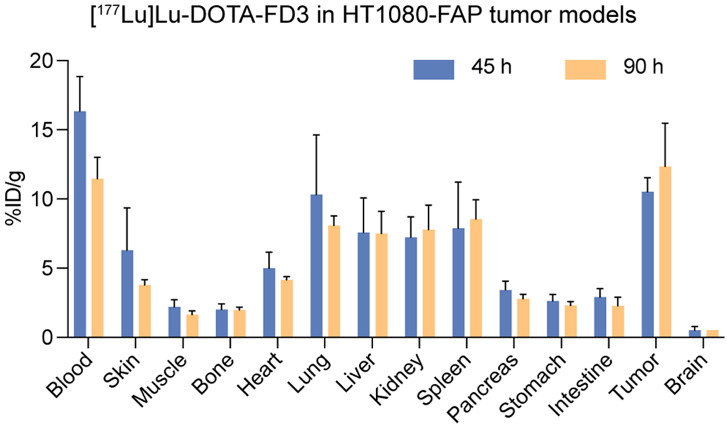


**Figure S10.** The radioactive biodistribution of [^177^Lu]Lu-DOTA-FD3 in HT1080-FAP tumor models at 45 h and 90 h after radiopharmaceuticals injection.


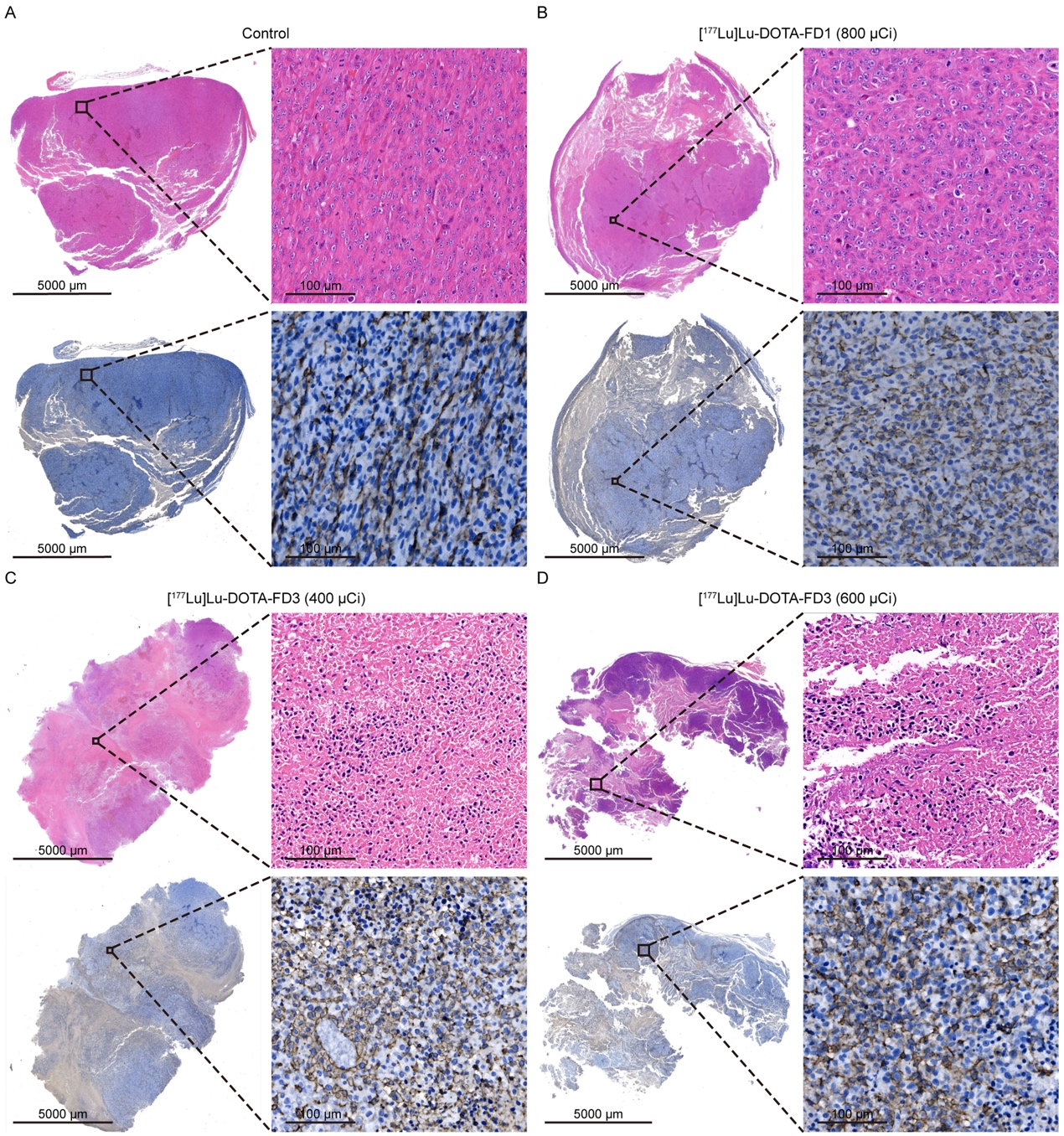


**Figure S11.** H&E and IHC results of tumor tissues from the HT1080-FAP models with an average tumor volume of 180 mm^3^ at the onset of the treatments. (**A–D**) H&E (upper) and IHC (lower) results of the tumor tissues from the relatively heavier tumor burden groups treated with PBS (**A**), 800 μCi [^177^Lu]Lu-DOTA-FD1 (**B**), 400 μCi [^177^Lu]Lu-DOTA-FD3 (**C**), and 600 μCi [^177^Lu]Lu-DOTA-FD3 (**D**).


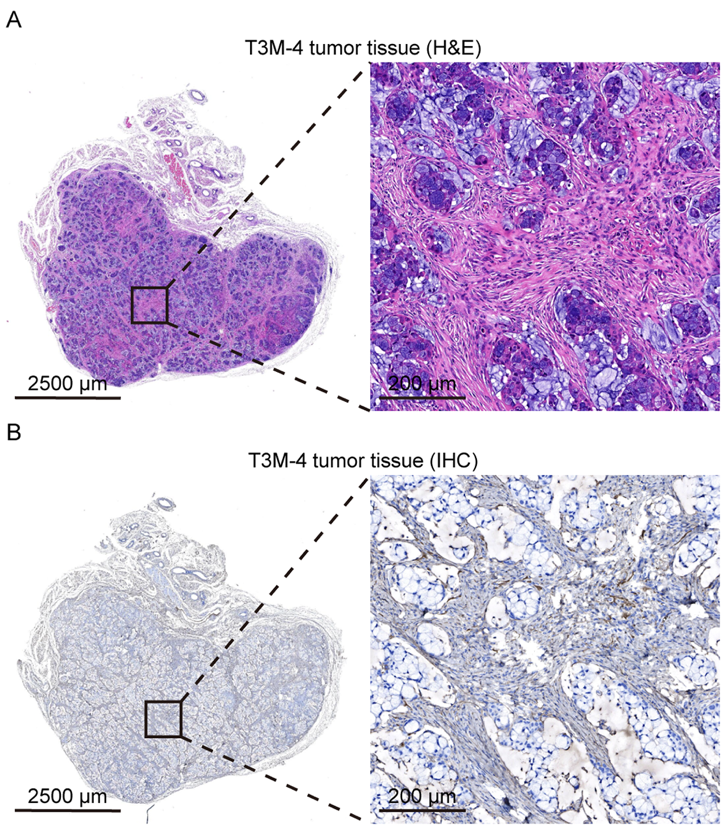


**Figure S12.** Hematoxylin-eosin (H&E) and IHC staining of T3M-4 tumor tissue. H&E (**A**) and IHC (**B**) staining showed FAP-expressing in the stroma.
